# Amphiphilic Fluorinated Unimer Micelles as Nanocarriers of Fluorescent Probes for Bioimaging

**DOI:** 10.1021/acsanm.3c02300

**Published:** 2023-08-28

**Authors:** Andrea Delledonne, Elisa Guazzelli, Silvia Pescina, Annalisa Bianchera, Giancarlo Galli, Elisa Martinelli, Cristina Sissa

**Affiliations:** † Dipartimento di Scienze Chimiche, della Vita e della Sostenibilità Ambientale, Università di Parma, Parco Area delle Scienze 17A, 43124 Parma, Italy; ‡ Dipartimento di Chimica e Chimica Industriale, Università di Pisa, 56124 Pisa, Italy; § ADDRes Lab, Dipartimento di Scienze degli Alimenti e del Farmaco, Università di Parma, Parco Area delle Scienze 27A, 43124 Parma, Italy; ∥ Centro per la Integrazione Della Strumentazione Dell’Università di Pisa (CISUP), Lungarno Pacinotti 43/44, 56126 Pisa, Italy

**Keywords:** amphiphilic polymer, unimer micelles, fluorescent nanocarriers, bioimaging, two-photon microscopy, fluorescence anisotropy, ex vivo permeation studies

## Abstract

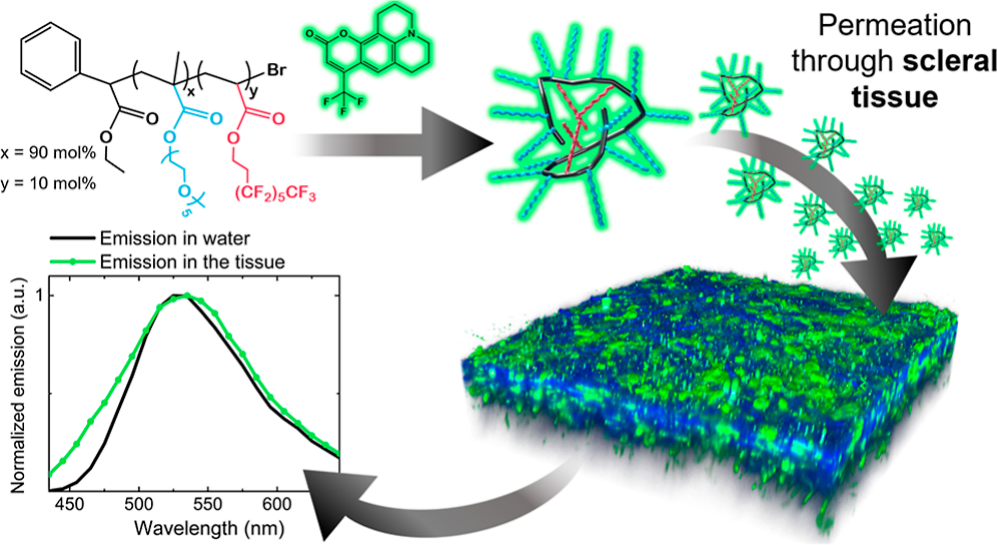

The unique self-assembly properties of unimer micelles are exploited for the preparation of fluorescent nanocarriers embedding hydrophobic fluorophores. Unimer micelles are constituted by a (meth)acrylate copolymer with oligoethyleneglycol and perflurohexylethyl side chains (PEGMA90-*co*-FA10) in which the hydrophilic and hydrophobic comonomers are statistically distributed along the polymeric backbone. Thanks to hydrophobic interactions in water, the amphiphilic copolymer forms small nanoparticles (<10 nm), with tunable properties and functionality. An easy procedure for the encapsulation of a small hydrophobic molecule (C153 fluorophore) within unimer micelles is presented. UV–vis, fluorescence, and fluorescence anisotropy spectroscopic experimental data demonstrate that the fluorophore is effectively embedded in the nanocarriers. Moreover, the nanocarrier positively contributes to preserve the good emissive properties of the fluorophore in water. The efficacy of the dye-loaded nanocarrier as a fluorescent probe is tested in two-photon imaging of thick ex vivo porcine scleral tissue.

## 1 Introduction

Several modern techniques for diagnostic imaging, bioassays, and biomarker analysis take advantage of the fluorescence of small organic molecules.^1−5^ Unfortunately, small organic fluorophores may suffer from limitations regarding photostability^6,7^ and aggregation-caused quenching^8^ in the biological environment. Moreover, organic fluorophores may exhibit toxicity and low water solubility, hindering the application of many promising molecules for in vitro and in vivo optical imaging. To overcome these limitations, the research on polymeric fluorescent micro/nanoparticles flourished, and a number of systems were proposed based on the copolymerization of monomers bearing fluorescent groups, conjugated polymers, or the decoration of non-fluorescent block copolymer micelles, dendrimers, and cross-linked polymer particles.^9−12^ Despite the improvement, the relatively large size of conventional polymeric particles (often ≫10 nm) still represents a shortcoming since it prevents efficient distribution and traversal of intact membranes. Moreover, in vivo accumulation of large particles in the body is a known critical point. For this reason, since their infancy, the so-called single-chain polymer nanoparticles (SCNPs) were endowed with fluorescent features,^13,14^ and their self-assembly in aqueous solutions was investigated in view of their potential use as fluorescent probes^15−20^ and carriers of therapeutics agents^21−24^ with improved biodistribution.^25^ Indeed, SCNPs are a convenient tool to obtain soft nanomaterials exploiting the folding of single polymer chains, via covalent or non-covalent intramolecular interactions. SCNPs can reach extremely small sizes (as small as 3 nm) as a function of molecular weight, functionalization degree, the nature of the interactions that drive the collapse, and the solvent quality.^26−28^ Among SCNPs, unimer micelles, generally formed by amphiphilic random copolymers in which the hydrophilic and hydrophobic comonomers are statistically distributed along the polymeric backbone, represent a straightforward option with convenient synthetic availability, that in some cases outperform well-known block copolymers self-assembly.^29−31^ By taking advantage of the hydrophobic interactions within the linear amphiphilic polymer, unimer micelles are able to form <10 nm nanoparticles,^32,33^ responsive to the external environment,^34−39^ with different domains of tunable polarity/affinity that can accommodate external molecules^40−45^ such as a fluorophore, even without the need of an additional synthetic step to functionalize the particle or complex encapsulation protocols. Moreover, unimer micelles are also thought to be stable at higher concentrations and to possess a more globular structure when compared to classical covalently crosslinked SCNPs.^16,46^

Fluorescence spectroscopy is a valuable tool for the investigation of dye-loaded nanocarriers. The interactions between the fluorophore and the nanocarrier^47^ and the interactions between multiple dyes loaded in the nanocarrier^48,49^ strongly affect the fluorescence response of the fluorophore itself, and this response can be exploited to retrieve information about the nanocarrier. More specifically, fluorescence solvatochromism can provide important information about the local environment of the fluorophore,^50^ while fluorescence anisotropy can be exploited to prove the fluorophore encapsulation and/or its mobility within the nanocarrier.^51,52^

**Figure 1:**
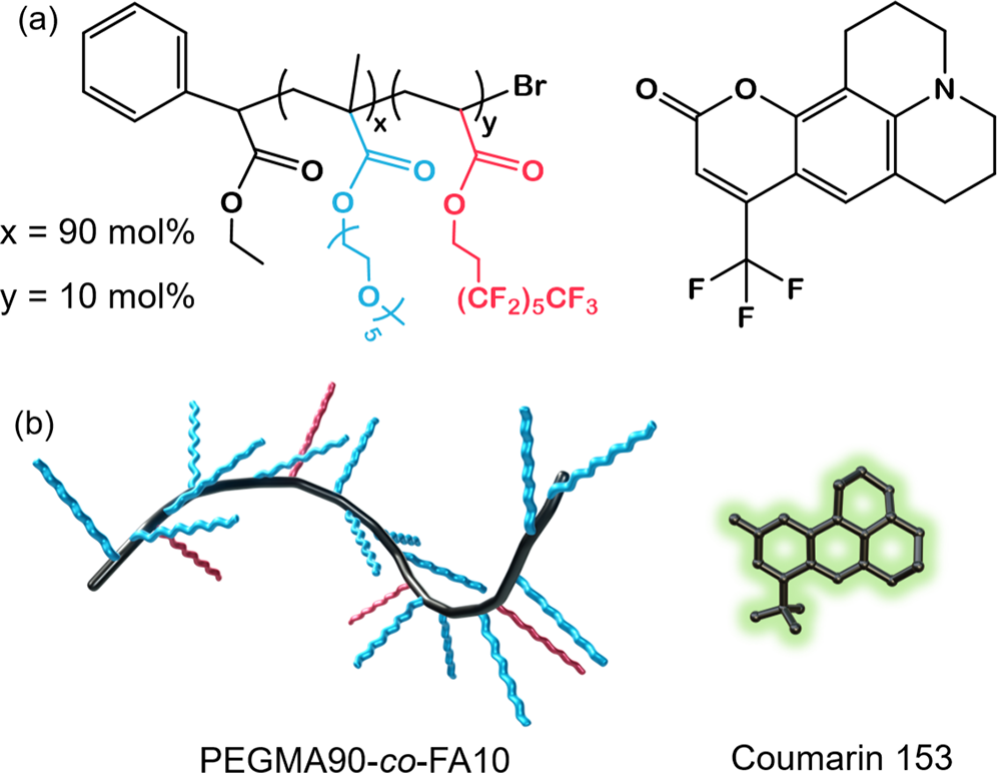
Molecular structures (a) and schematic representation (b) of PEGMA90-*co*-FA10 (*x* = 90, *y* = 10 mol %) and Coumarin 153 (C153).

In this study, an amphiphilic random (meth)acrylic copolymer with oligoethyleneglycol and perflurohexylethyl side chains (PEGMA90-*co*-FA10) was chosen to encapsulate Coumarin 153 (C153) (Figure 1) to be used as a fluorescent nanotool for the topical application (i.e., skin and mucous membranes, such as ocular). In particular, the selected copolymer was characterized by a relatively small content of hydrophobic FA counits to ensure its complete solubility in water, but sufficient to promote the copolymer self-assembly in water into unimer micelles, as already demonstrated by dynamic light scattering (DLS) and small-angle neutron scattering (SANS) measurements in a previous work by some of us.^35^ The self-folding of the copolymer is crucial for its functional application as a nanocarrier for a hydrophobic fluorophore, namely, C153. On the other hand, C153, widely reported as a fluorescent probe for different applications, including bioimaging^53−55^ and investigation of micellar systems,^56−58^ was selected because of (i) its small molecular dimensions, an important feature for studying the encapsulation via fluorescence anisotropy studies; (ii) hydrophobicity, favoring the encapsulation in the hydrophobic compartment of unimer micelles; (iii) good fluorescence quantum yield, which is required for bioimaging; and (iv) its solvatofluorochromic behavior,^58^ allowing for the investigation of the local microenvironment of the fluorescent probe. In addition, C153 was also previously adopted as a probe for measuring time-resolved fluorescence anisotropy,^59,60^ which provides important information about the mobility of the dye interacting with the nanocarrier.^51,52,61^

C153-loaded unimer micelles are carefully characterized to investigate the dye encapsulation and the emissive properties of the fluorescent nanocarrier. The efficiency of the C153-loaded unimer micelles as fluorescent probes is tested in multiphoton microscopy by visualizing ex vivo scleral tissue of a porcine eye. Multiphoton microscopy is a powerful tool for the in-depth imaging of biological tissues,^62^ and recently, it has been applied to drug delivery studies to follow the fate of the nanocarrier.^73,74^ One of the major advantages of multiphoton microscopy with respect to confocal fluorescence microscopy is the possibility to reconstruct 3D images with a penetration depth up to 1–2 mm, requiring the efficient permeation of the fluorescent probe.

In this paper, we present a novel application of spontaneously self-folded unimer micelles of amphiphilic random copolymers as nanocarriers for a hydrophobic fluorescent probe. More generally, the work represents a proof of concept of the ability of self-folded unimer micelles to transport small hydrophobic molecules, avoiding covalent interactions, and with potential applications also in the fields of drug delivery, sensing, etc. We propose a process for the production of fluorescent unimer micelles that is simple, and less chemically demanding, compared to the formation of fluorescent SCNPs by multistep intramolecular crosslink.^15,18,21,23,40^ Then, we demonstrate the efficient permeation in sclera of the dye-loaded unimer micelles, allowing the direct visualization of the fluorescent probe in the whole tissue.

## 2 Experimental Section

### 2.1 Synthesis

#### 2.1.1 Materials

Anisole (Sigma-Aldrich) was vacuum distilled over sodium. CuBr (Sigma-Aldrich) was extracted with glacial acetic acid and then washed with diethyl ether, dried, and stored under nitrogen. *N*,*N*,*N*′,*N*″,*N*‴-Pentamethyldiethylenetriamine (PMDETA, Sigma-Aldrich) and ethyl α-bromophenylacetate (EBPA) were freshly distilled before use. Perfluorohexylethyl acrylate (FA, Fluorochem) and PEGMA (*M*_n_ = 300 g mol^–1^, DP_n_ ≈ 5, *D̵* = 1.2, Sigma-Aldrich) were filtered on basic alumina to remove inhibitors.

#### 2.1.2 Synthesis of PEGMA90-*co*-FA10

The copolymer PEGMA90-*co*-FA10 was prepared and purified according to an already established procedure.^33,63^ Briefly, PEGMA (2.60 g, 8.7 mmol), FA (0.418 g, 1.0 mmol), PMDETA (20.1 μL, 0.1 mmol), EBPA (16.9 μL, 0.1 mmol), and anisole (6 mL) were degassed in a Schlenk tube with three freeze–pump–thaw cycles. Then, CuBr (14.34 mg, 0.1 mmol) was added and three more freeze–pump–thaw cycles were performed before the polymerization was carried out at 90 °C under a nitrogen atmosphere for 24 h. The reaction was stopped by exposure to air and the crude product was filtered on basic alumina to remove the catalyst and repeatedly precipitated from chloroform into *n*-hexane (75% yield). The copolymer was characterized by ^1^H and ^19^F NMR (Figure S1) and GPC in CHCl_3_ (Figure S2). The copolymer contained 90 mol % PEGMA and 10 mol % FA counits from ^1^H NMR analysis with a resulting *M*_n_ = 21,000 g mol^–1^.

^1^H NMR (acetone-*d*_6_): δ (ppm) 7.2–7.4 (arom), 4.0–4.5 (COOCH_2_), 3.4–3.8 (OCH_2_CH_2_), 3.3 (OCH_3_), 2.5–2.8 (CH_2_CF_2_), 1.5–2.3 (CH_2_CH), 0.8–1.4 (CH_3_).

^19^F NMR (CDCl_3_/CF_3_COOH): δ (ppm) −5.1 (CF_3_), −37.9 (CF_2_CH_2_), −46.1 to −48.0 (CF_2_), −50.4 (CF_2_CF_3_).

GPC (CHCl_3_/PMMA): *M*_n_ = 4600 *D̵* = 1.21

#### 2.1.3 Characterization

^1^H NMR and ^19^F NMR solution spectra were recorded with a Bruker Avance DRX 400 spectrometer. The number and weight average molecular weights and dispersity (*M*_n_, *M*_w_, and *D̵*) were determined by gel permeation chromatography (GPC) using a Jasco PU-2089 Plus liquid chromatograph equipped with two PL gel 5 μm mixed-D columns, a Jasco RI-2031 Plus refractive index detector, and a Jasco UV-2077 Plus UV/vis detector. Measurements were carried out using chloroform as the mobile phase, at a flux of 1 mL/min and a temperature of 30 °C maintained by a Jasco CO 2063 Plus column thermostat. Samples were filtered with a 0.2 μm PTFE filter before injection. Poly(methyl methacrylate) standards were used for calibration.

### 2.2 Micelles Preparation and Loading

Dye-loaded unimer micelles were prepared adopting the thin-film re-hydration procedure. The copolymer PEGMA90-*co*-FA10 and the dye C153 (Sigma-Aldrich) were first dissolved in acetone. The solvent was then completely removed under vacuum obtaining a thin film. An appropriate volume of distilled water or saline solution was added to obtain the final concentrations of 5 g/L (∼238 μM) and 15 μM for PEGMA90-*co*-FA10 and C153, respectively. A different preparation procedure of dye-loaded unimer micelles was tested for some control experiments: a small amount of a concentrated C153 solution in acetone was added (final acetone concentration in the suspension: ∼0.1% v/v) to a suspension of PEGMA90-*co*-FA10 in water (5 g/L) obtaining a final C153 concentration of 5 or 30 μM. All the obtained polymeric suspensions (prepared with the two procedures) were magnetically stirred for 1 h and then filtered (hydrophilic PTFE, AISIMÔ 0.22 μm). The control solution of C153 in distilled water employed for permeation experiments was obtained following the thin-film re-hydration procedure without adding the polymer. In the case of the control solution, the total theoretical concentration of C153 was 30 μM, but the final concentration after the filtration (hydrophilic PTFE, AISIMÔ 0.22 μm) is limited by the low water solubility of the dye. The C153-saturated solutions in distilled water or saline solution used for spectroscopic measurements were prepared by adding 0.5 g/L of C153 to the solvent, magnetically stirring for 1 h, and then filtering the resulting suspension (hydrophilic PTFE, AISIMÔ 0.22 μm).

### 2.3 Spectroscopic Characterization

Solutions for spectroscopic measurements in acetone and 2-MeTHF were prepared using spectrophotometric or HPLC-grade solvents. Polyethylene glycol 400 (PEG400) is of pharmaceutical quality according to Ph.Eur.-USP. UV–vis absorption measurements were performed with a PerkinElmer Lambda650 spectrophotometer, while both emission spectra and fluorescence anisotropies were recorded with a FLS1000 Edinburgh fluorometer, equipped with automatic polarizers. Fluorescence quantum yields of all the samples were measured using fluorescein in NaOH_aq_ 0.1 M as the standard (ϕ = 0.9).

Anisotropy measurements at 77 K were collected using an Oxford Instrument OptistatDN cryostat by rapidly cooling down 2-MeTHF (stored over molecular sieves for one night, and filtered before use), obtaining a glassy matrix. Fluorescence anisotropy (*r*) is measured by exciting the sample with linearly polarized light and collecting the emission with parallel (*I*_VV_) and orthogonal (*I*_VH_) polarization with respect to the exciting beam. Fluorescence anisotropy is defined as follows
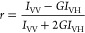
where *I* is the emission intensity, and the subscripts V/H represent the orientation of the excitation and emission polarizers, respectively. The *G* factor correction accounts for the different sensitivities of the detector to the vertical and horizontal polarization of the emitted light and it is defined as
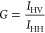


Steady-state excitation anisotropy spectra are recorded as a function of the excitation wavelength and detecting the emission at a fixed frequency.

Lifetime decays and time-resolved anisotropy measurements were acquired by exciting the sample with a pulsed diode laser (∼200 ps pulse duration and 405 nm as excitation wavelength) at a repetition rate of 1 MHz.

### 2.4 Dynamic Light Scattering

DLS measurements were performed at 25 °C with a Malvern Zetasizer Nano ZSP apparatus equipped with a 633 nm HeNe laser (Malvern Instruments, Malvern, UK). The backscattering mode (scattered light is collected at an angle of 173°) was employed for all the analyzed samples.

### 2.5 Ex Vivo Permeation Experiments across Scleral Samples

Scleral samples were isolated from fresh pig eyes (breed: Landrace and Large White; sex: female and male animals; weight: 145–190 kg; age: 10–11 months; provided by a local slaughterhouse) as previously described.^64^ The tissue was mounted on a glass Franz-type diffusion cell (DISA, Milano, Italy) with a permeation area of 0.6 cm^2^, with the episclera (i.e., the outer side) facing the donor chamber, and the choroidal side (i.e., the inner surface) in contact with the receiving chamber. The receiving chamber was filled with saline solution (NaCl 9 g/L) previously degassed (4 mL, exactly measured); the solution was magnetically stirred during the experiment to guarantee sink conditions and kept at 37 °C. The donor chamber was filled with 200 μL of the dye-loaded polymeric formulation applied without dilution at infinite dose. Two additional scleral sample tissues were mounted on Franz-type cells filling the donor with pure saline solution (NaCl 9 g/L) or with the control solution containing C153 in distilled water (described in paragraph 2.2). After 2 h, scleral samples were visualized via two-photon microscopy (Sections 2.6 and 3.2).

### 2.6 Two-Photon Microscopy

Ex vivo porcine scleral samples were analyzed with a Two-Photon Microscope Nikon A1R MP+ Upright equipped with a femtosecond pulsed laser Coherent Chameleon Discovery (∼100 fs pulse duration with 80 MHz repetition rate, tunable excitation range 660–1320 nm). A 25× water dipping objective with numerical aperture 1.1 and 2 mm working distance was employed for focusing the excitation beam and for collecting the two-photon excited fluorescence (TPEF) and the second harmonic generation (SHG) signals. TPEF/SHG signal was directed by a dichroic mirror to a series of three non-descanned detectors (high sensitivity GaAsP photomultiplier tubes) enabling fast image acquisition. The three detectors were preceded by optical filters, allowing the simultaneous acquisition of three separated channels: blue channel (415–485 nm), green channel (506–593 nm), and red channel (604–679 nm). Imaging overlay of the three channels and processing was performed by the operation software of the microscope. Additionally, a fourth GaAsP photomultiplier detector, connected to the microscope through an optical fiber and preceded by a dispersive element, was used to record the spectral profile of the TPEF/SHG signal (wavelength range 430 to 650 nm with a bandpass of 10 nm). Scleral discs were placed in a dedicated plexiglass holder, right after dismounting the tissue from the Franz-type cell and using saline solution to dip the objective and to avoid dehydration. Images were acquired exciting the tissues at 830 nm with a typical field of view of 500 μm × 500 μm, except where explicitly reported.

### 2.7 In Vitro Toxicity Tests

The effect of the polymer on cell viability was evaluated as effect on mitochondrial activity by using the 3-(4,5-dimethylthiazol-2-yl)-2,5-diphenyltetrazolium bromide (MTT) assay on HEK 293 cell line (ATCC CRL-1573), and epithelial carcinoma cell line, A549 (ATCC CCL-185). After expansion, cells were seeded into 96-well plates (VWR Tissue Culture Plates, VWR International, Italy) at a density of 6 × 10^4^ cells/well for HEK 293, or 1 × 10^4^ cells/well for A549, in a culture medium composed of DMEM (Dulbecco’s minimum essential medium, Biowest, Nuaillé, France) with the addition of 10% fetal bovine serum (FBS, Heat inactivated, Thermo Fisher Scientific, Waltham, MA, USA), 1% penicillin/streptomycin solution, and 1% of nonessential amino acid solution (MEM NEAA) all obtained from Gibco (Thermo Fisher Scientific, Waltham, MA, USA). Cells were left to settle overnight before performing the viability assay.

PEGMA90-*co*-FA10 was dissolved in the culture medium to a final concentration of 20 g/L and further diluted in the same medium, with serial two-fold dilutions to get a range of concentrations between 0.04 and 10 g/L of polymer. Before the test, the growth medium was removed and 100 μL of each solution to be tested was added to each well and left for 3 h at 37 °C and 5% CO_2_. After incubation, solutions were gently removed and 100 μL of 1 g/L solution of MTT (M2128, Sigma-Aldrich, St Louis, MO, USA) in DMEM was added and left for 3 h at 37 °C and 5% CO_2_. After removing the solution, precipitated formazan crystals were dissolved in 100 μL of DMSO for each well, with shaking, for 10 min in the dark. Then, the absorbance of the samples was read at 570 nm employing a plate reader Spark (Tecan, Männedorf, Switzerland). The viability of cells was expressed as a percentage with respect to untreated control as mean value ± standard deviation (*n* = 5).

## 3 Results and Discussion

### 3.1 Self-Assembly of Unimer Micelles

The amphiphilic random (meth)acrylic copolymer PEGMA90-*co*-FA10 (Figure 1) was synthesized by atom transfer radical polymerization (ATRP) of hydrophobic perfluorohexylethyl acrylate (FA) with a hydrophilic oligo(ethylene glycol) methyl ether methacrylate containing ∼5 repeating units (PEGMA) following a literature procedure^33,63^ to obtain a water-soluble copolymer with a large hydrophilic character (90 %mol PEGMA units, *M*_n_ = 21,000 g mol^–1^ by ^1^H NMR, see Figure S1) and with a narrow, monomodal molecular weight distribution (*D̵* = 1.21 from GPC analysis, see Figure S2).

The self-assembly behavior in an aqueous solution of amphiphilic random copolymers with perfluoroalkyl and oligooxyethylene side chains such as the copolymer PEGMA90-*co*-FA10 used in this work was previously investigated in great detail as a function of copolymer concentration and temperature by different and complementary techniques, including DLS, NMR relaxometry, SANS and SAXS analyses, and integrated with molecular dynamics simulations.^11,33,35,39,63^ In particular, SANS profiles of copolymer PEGMA90-*co*-FA10 in D_2_O at different concentrations (1–10 g/L) and as a function of temperature demonstrated the existence of non-aggregated single polymer chains with an *R*_g_ ∼2 nm (from an ellipsoidal fitting) below 45 °C. The bell-shaped Kratky plots elaborated from SANS data demonstrated, that independently of copolymer concentration, the single-chain unimers exhibited a self-folded, compact and low flexible, globular conformation with size (i.e., pseudo-Guinier radius, *R*_pg_) of ≈3 nm. The self-folding of the macromolecular chains is driven by the hydrophobic intramolecular interactions of the fluorinated side chains when the copolymer is dissolved in water.^35^ Such a self-assembly mechanism was also supported by computational studies of the folding trajectory of a typical PEGMA-*co*-FA copolymer in water in terms of decrease in *R*_g_, especially when compared to the size of the unfolded copolymer in a non-selective organic solvent, and the evolution of the solvent-accessible surface area. The latter in particular demonstrated a general reduction of the surface exposed to the selective solvent (water), which was mainly related to the FA component gradually becoming less and less exposed to the aqueous environment.^33^

**Figure 2:**
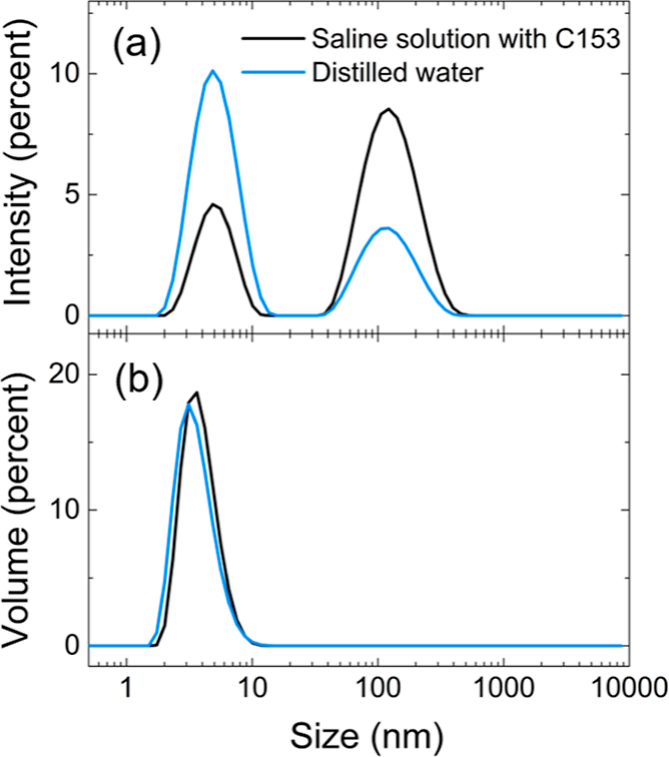
Intensity [panel (a)] and volume [panel (b)] size distributions of the unimer micelles in distilled water without dye (blue lines, total polymer concentration: 5 g/L) and in saline solution after loading with C153 (black lines, total polymer concentration: 5 g/L, total dye concentration: 15 μM).

The advantage of using the folding of a single-chain object rather than a supramolecular aggregate is the formation of unimer micelles with exceptionally low size, being the diameter *D*_h_ generally <8 nm and the gyration radius *R*_g_ ∼2 nm; it can also display additional features such as thermoresponsive aggregation. Moreover, unimer micelles were shown to be able to encapsulate organic molecules, that were useful to probe the formation of segregated hydrophobic domains within the micelle itself via their fluorescence emission.^11,33^ Recently, the incorporation and controlled release of a highly hydrophobic drug (combretastatin A-4) in PEGMA-*co*-FA unimer micelles has been achieved as a proof of concept of their application in the biomedical field. On the basis of these results, PEGMA90-*co*-FA10 unimer micelles were loaded with C153, following the procedure described in Section 2.2. C153-loaded unimer micelles were prepared both in water and in saline solution in order to have an appropriate medium for biological applications. The self-assembly was verified by DLS at room temperature. Figure 2 shows the size distributions obtained from DLS measurements for the unloaded copolymer in distilled water and for the C153-loaded unimer micelles in saline solution (the concentration of the copolymer is the same in the two samples): the intensity size distribution shows two populations for both samples, one having a *D*_h_ of ∼5 nm and the other of ∼130 nm. The presence of a population having *D*_h_ ∼5 nm is consistent with the formation of unimer micelles in both samples as expected from the previous studies on self-folding of PEGMA-*co*-FA analogue copolymers.^35,39,63^ A second population of larger particles (*D*_h_ ∼ 130 nm) is also usually detected in the intensity distribution,^33,63^ but it is absent in the volume distribution (Figure 2b) indicating a negligible tendency of the copolymer to self-assemble into larger multi-chain aggregates. Although negligible, such a contribution is more pronounced for the saline solution containing C153 as the relative intensity of the associated peak is higher (Figure 2a), suggesting that the presence of the C153 dye and/or a change in ionic force promotes the formation of aggregates. The presence of C153 might favor the aggregation of different polymeric chains acting as a physical cross-linker, as was already observed after the loading of a hydrophobic drug^24^ and a fluorinated agrochemical^41^ in similar systems. Moreover, it was recently shown that NaCl aqueous solutions in the range of the physiological concentration favor the dehydration of the oxyethylene side chains, which results in a reduced solubility of the copolymer, with a slight decrease in the value of the solution cloud point.^39^ The negligible contribution of the multi-chain aggregates with respect to that of unimer micelles was also previously demonstrated by SANS measurements of PEGMA90-*co*-FA10 solution in D_2_O that showed only the presence of unimer micelles at relatively low temperatures (<45 °C). By increasing the temperatures above 45 °C the self-folded unimers started to aggregate in larger multi-chain particles, with a minimal weight fraction of larger aggregates being detectable at 45 °C (2 wt %) and gradually increasing up to 98 wt % at 60 °C.^35^ The volume size distributions do not show any significant difference and clearly indicate that neither C153, nor NaCl (9 g/L) significantly impact the structure of unimer micelles (see Table S1 and Figure S3 for a summary of DLS results obtained with all the prepared polymeric suspensions).

### 3.2 C153 Dye Encapsulation in Unimer Micelles

#### 3.2.1 UV–Vis Spectroscopy in Aqueous Media

**Figure 3:**
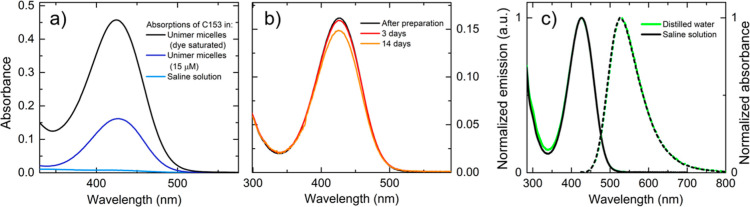
(a) Comparison between the absorption spectra of C153-loaded unimer micelles (polymer concentration: 5 g/L, total theoretical dye concentration: 15 μM), C153 saturated unimer micelles (polymer concentration: 5 g/L, total theoretical dye concentration: 0.5 g/L), and C153-saturated saline solution. (b) Absorption overtime variations of C153-loaded micelles. (c) Comparison between normalized absorption and emission spectra of C153-loaded unimer micelles in distilled water and saline solution.

C153 is a strongly hydrophobic dye, and its solubility in water is very poor:^59^ the maximum absorbance obtained in C153-saturated saline solution is 0.008 at 431 nm (maximum of the low-energy absorption band of C153 in water), as reported in Figure 3a. In the presence of the copolymer (total polymer concentration: 5 g/L), for a relatively low dye concentration of 15 μM (theoretical concentration), the absorbance of the suspension is significantly increased and reaches the value of 0.15, also maintaining a good stability over time up to 2 weeks after preparation (Figure 3b, corresponding DLS results reported in Figure S5). More absorptive solutions can be obtained at higher concentration of dye, as shown in Figure 3a (Figure S4 shows the results obtained with different preparation methods). Further spectroscopic characterizations (and two-photon microscopy experiments) were performed on suspensions having a polymer concentration of 5 g/L (which is a good concentration for DLS experiments), and a low dye concentration (15 μM) to avoid inner filter effects in fluorescence experiments and the formation of dimers or aggregates in the micelles, which could affect the spectroscopic properties of the fluorophore.

UV–Vis and fluorescence spectra (Figure 3c), as well as lifetime decays (Table S2) of dye-loaded micelles prepared in water and in saline solution are superimposable; thus, the spectroscopic characterization discussed further in the text is referred to the suspension in distilled water. The presence of the copolymer significantly contributes to preserve the good emissive properties of C153: the fluorescence quantum yield of C153-loaded micelles is 50%, a value comparable to those measured in other organic solvents.^58,65^ The fluorescence lifetime (τ) measured in the unimer micelles suspension is significantly higher than the values obtained from C153 in water or saline solution (Table 1, Figure S6, and Table S2). These results confirm that unimer micelles are able to efficiently solubilize the C153 dye in an aqueous medium, preserving its good emissive properties.

**Table 1:** Spectroscopic Properties of C153 in Different Environments

	λ_abs_^max^ (nm)	λ_em_^max^ (nm)	Stokes shift (cm^–1^)	fluorescence quantum yield ϕ (%)	fluorescence lifetime τ (ns)^b^
perfluorohexane	383	438	3279	68	4.1
acetone	417	515	4563	78	5.6
PEG400	425	530	4661	68	4.9
unimer micelles in distilled water	425	530	4661	50	4.6
distilled water	431	541	4718	14^a^	1.9

a The quantum yield value measured in distilled water is in good agreement with the one reported in ref (65) and similar to the one obtained from C153 saline solution (13%).

b Further details about the lifetimes and the fitting results are reported in Figure S6 and Table S2.

#### 3.2.2 Fluorescence Anisotropy Studies

**Figure 4:**
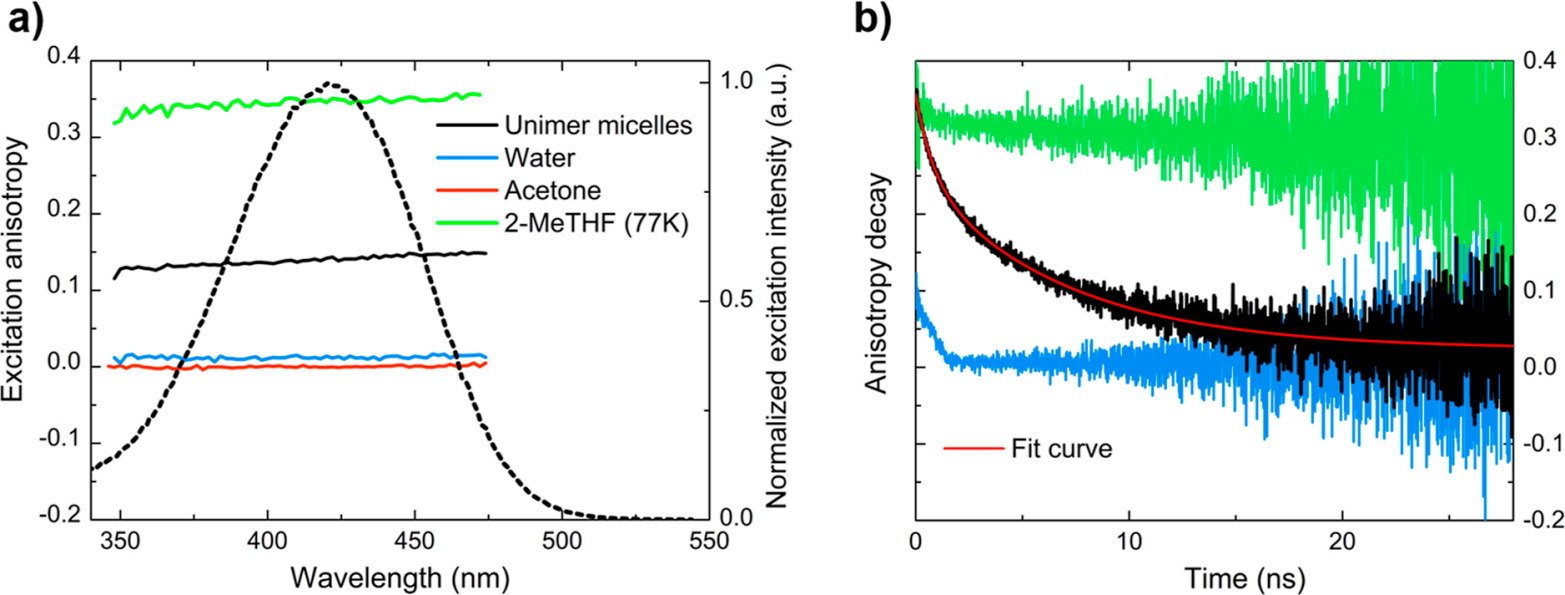
(a) Steady-state excitation anisotropy of C153 in different environments (λ_em_ = 550 nm). (b) Time-resolved anisotropies of C153 in different environments measured by exciting the sample at 405 nm and detecting emission at 500 nm (2-MeTHF at 77 K and unimer micelles) or 515 nm (water). The red solid line represents the best fitting of eq 3 to the experimental fluorescence anisotropy decay obtained from C153-loaded unimer micelles.

Fluorescence anisotropy is a powerful tool to investigate the change of the environment when a small fluorescent molecule is embedded in a nanocarrier. Fluorescence anisotropy (*r*) is related to the rotational correlation time (τ_c_) and the fluorescence lifetime (τ) through the Perrin equation
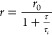
1where *r*_0_ is the intrinsic anisotropy of the dye that is measured in the absence of diffusion.^66^ The intrinsic anisotropy *r*_0_ of C153 was measured in 2-MeTHF transparent glass at 77 K and resulted equal to 0.35 (Figure 4a), very close to the limiting value of anisotropy 0.4, in agreement with data reported in the literature.^67^ According to eq 1, in non-viscous solvents such as water or acetone, the rotational correlation time is much shorter than the emission lifetime of C153, and the Brownian motions of the molecule depolarize molecular fluorescence, resulting in anisotropy value close to zero (Figure 4a). The anisotropy measured from the C153-loaded unimer micelle solution amounts to ∼0.14 (Figure 4a), confirming that C153 is embedded in the micelles, which have a correlation time much higher with respect to the molecule due to their bigger dimensions. However, the anisotropy value of C153 measured from unimer micelle suspension is affected not only by the motion of the micelles but also by the motion of C153 within the micelles, and the separation of these two contributions is not trivial. From eq 1, we calculated an effective orientational correlation time of 3 ns for the C153 embedded unimer micelles. The τ_c_ of a particle is defined as the time needed for a rotation of 1 radian and it is related to the hydrodynamic volume (*V*_h_) by the following equation
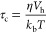
2where η is the medium viscosity, *T* is the temperature, and *k*_b_ is the Boltzmann constant. Assuming a perfect spherical shape, the correlation time associated with the hydrodynamic diameter determined by DLS measurements (5 nm) can be estimated from eq 2 and amounts to 16 ns. As expected, the rotational correlation time obtained by steady-state fluorescence anisotropy is lower than the rotational correlation time derived from DLS. The former is underestimated because the internal motion of the dyes loaded in the nanoassemblies further contributes to the depolarization of the emitted light.

Additional information concerning the rotational motion of the fluorescent probe can be extracted from time-resolved anisotropy decays reported in Figure 4b. In the case of C153 in vitrified 2-MeTHF at 77 K, the *r* value starts at 0.35 and remains constant during the entire fluorophore decay (up to ∼20 ns), as expected due to the hindered rotational diffusion in this matrix. The anisotropy decay of C153 in water starts from a low value (∼0.1) and rapidly decreases, pointing to fast depolarization even before the earliest accessible time with our experimental setup. When the fluorescent probe is loaded in the unimer micelles the rotational motion is partially hindered (if compared to C153 in liquid solution) and the resulting anisotropy decay reaches a steady value slightly higher than zero (*r* = 0.02) after ≈20 ns. This residual anisotropy (*r*_∞_) suggests that the rotational diffusion angular range of C153 in its local environment is limited.^66^ The experimental anisotropy decay measured from the dye-loaded unimer micelles suspension is well described with a model that accounts for restricted rotational motion (wobbling-in-cone model) and translational diffusion of the dye coupled with the rotation of the whole micelle.^61^ Assuming that only the encapsulated C153 contributes to the recorded anisotropy, a bi-exponential function was employed to fit experimental data (Figure 4b, red line)
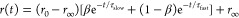
3where τ_slow_ and τ_fast_ are time constants, respectively, of 6.9 and 0.89 ns, associated to two different kinds of motions, both contributing to the orientational diffusion of the fluorescent probe. The parameter β (= 0.69 in our fit) represents the fractional contribution of the slower motion. The optimized values of *r*_0_ and *r*_∞_ amount to 0.35 and 0.02, respectively. Additional data such as the simultaneous fitting of the polarized fluorescence decays are reported in Section 1.8 of the Supporting Information (Figure S9). The larger time constant (τ_slow_ = 6.9 ns) extracted from the fitting procedure can be assigned to the orientational diffusion of the nanocarrier as a whole, together with the lateral diffusion of the encapsulated dye along the curved surface of the micelle. The presence of the latter fluorescence depolarization mechanism justifies why τ_slow_ is significantly lower than the expected correlation time for the unimer micelles, obtained from eq 2 and DLS results (16 ns). The shorter time constant (τ_fast_ = 0.89 ns) is mainly associated with the restricted wobbling motion of C153 in its local environment, with perturbations from both the orientational diffusion of the whole micelle and, once again, the translational diffusion of the dye. We underline that the time constants resulting from the fitting procedure are effective quantities since the employed model function assumed the unimer micelles to be spherical, without accounting for their prolate shape.^63^

#### 3.2.3 Solvatochromic Studies

**Figure 5:**
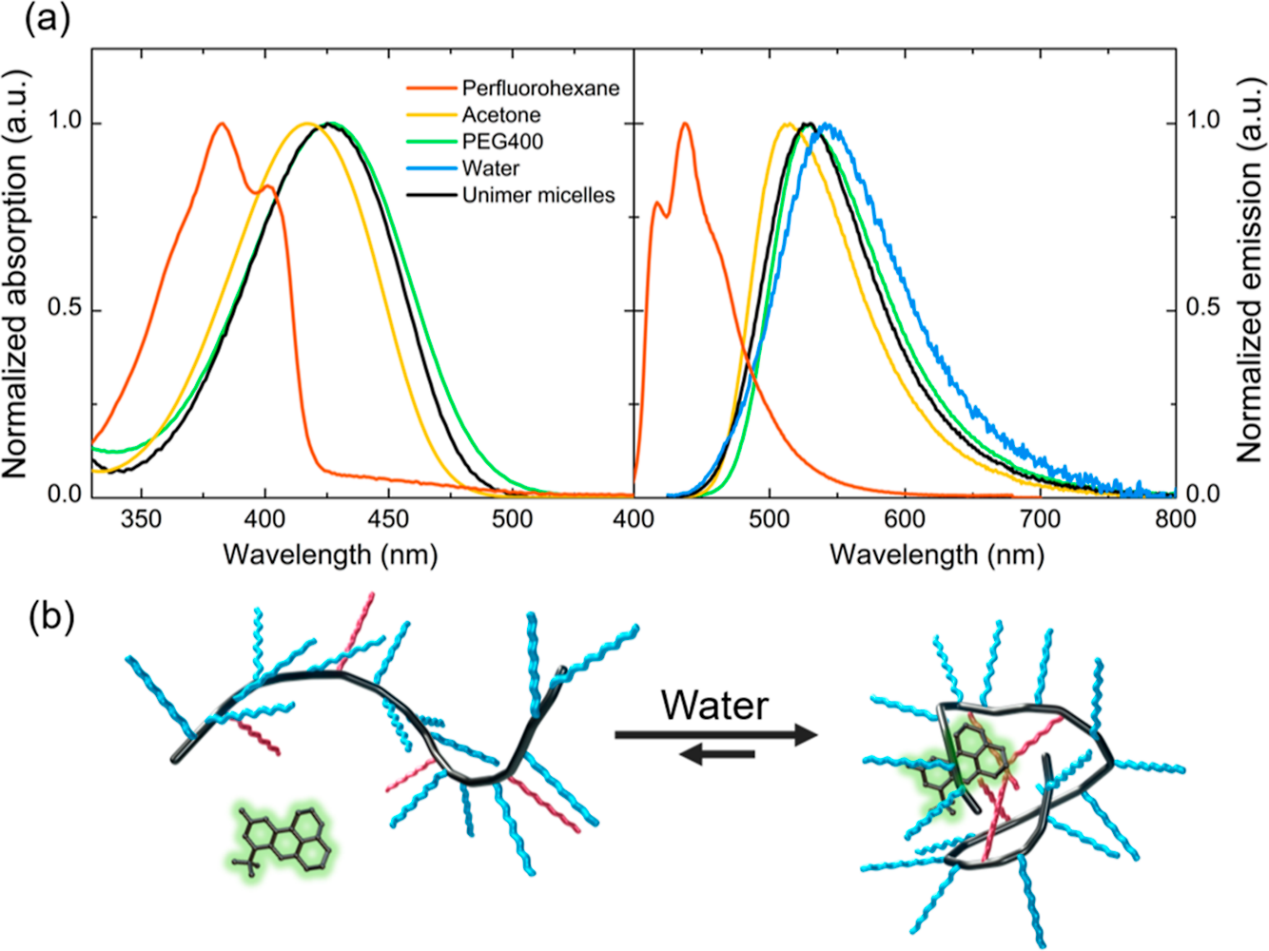
(a) Comparison between normalized absorption and emission spectra of C153 in different environments (the absorption spectrum in water is not reported for clarity). The emission spectra are acquired using an excitation wavelength of 420 nm for acetone, PEG400, water and for the suspension of unimer micelles, and 380 nm for perfluorohexane solution (the absorbance of solutions/suspensions is lower than 0.1). (b) Schematic representation of the self-folding mechanism of a copolymer chain in water in the presence of C153 dye.

Further information about the dye encapsulation can be retrieved from absorption and emission spectra of C153 collected in different solvents. C153 is a solvatochromic dye and both the emission and absorption spectra shift to longer wavelengths when the polarity of the medium is increased, as a consequence of an intramolecular charge transfer transition.^56,58^Figure 5 compares absorption and emission spectra of C153-loaded unimer micelles with spectra of C153 in perfluorohexane, acetone, PEG400, and water. Perfluorohexane is the nonpolar solvent that better mimics the hydrophobic core of the unimer micelles (FA units). Acetone is the polar organic solvent in which C153 is co-solubilized with the copolymer for the preparation of unimer micelles by re-hydration, while PEG400 was selected to mimic the oxyethylenic side chains of the copolymer that are located in the outer shell of the unimer micelles, at the interface with water. The emission and absorption profiles of the C153-loaded unimer micelles suspension (black line in Figure 5) are blue-shifted compared to spectra collected in water, confirming their encapsulation in the micellar system. The spectra of C153 loaded in unimer micelles are similar to the spectra collected in pure PEG400, and their spectral position is intermediate between those in acetone and water. This result indicates that C153 is located in a less polar environment than water, but still quite polar being it comparable to that of non-hydrated PEG. Unimer micelles are very complex systems in terms of local polarity and the precise localization of C153 is difficult to assess. In fact, although during the single-chain folding the perfluoroalkyl chains tend to be buried in the core of the unimer micelles,^33^ the existence of conformational and compositional constraints make their location in the inner compartment preferential, but not exclusive and PEG segments can also be present. Moreover, when adding a concentrated solution of C153 in acetone to a water suspension of self-assembled unimer micelles, we obtained superimposable UV–vis and emission spectra to those prepared by re-hydration, suggesting that the environment of C153 is the same, despite the different preparation procedure (the corresponding DLS results are reported in Table S1 and Figure S3, while the absorption and emission spectra are shown in Figure S8).

### 3.3 Unimer Micelles as Nanocarriers of Fluorescent Probes for Bioimaging

Fluorescence microscopy techniques reconstruct the image of a sample through the detection of fluorescence signals. Fluorescence can be emitted by the sample itself, due to the presence of endogenous fluorophores (autofluorescence), or by a fluorescent probe which is added to the sample through a staining procedure. One of the major issues concerning the use of fluorescent probes is their compatibility with the biological environment, i.e., the solubility and the preservation of their emissive properties in aqueous media.

In this work, we tested the efficacy of C153-loaded unimer micelles as fluorescent nanoprobes for two-photon microscopy (2PM). 2PM is a powerful imaging technique that allows in-depth imaging, up to 1–2 mm, of biological tissues,^62,68^ and hence requires an efficient permeation of the fluorescent probe. Technical details about sample preparation and the microscopy technique are reported in the Experimental Section. Ex vivo porcine scleral tissue was selected as testing biological material, being a robust recognized animal model for the study of the trans-scleral diffusion of drug addressing the posterior segment of the eye.^69^ Porcine sclera, similar to the human one, is mainly composed of randomly packed collagen fibers embedded in a proteoglycan matrix and traversed by elastin fibers and fibroblasts; the water content is approx. 70%.^69^ Apart from its crucial physiological and mechanical functions, this tissue was selected to test the C153-loaded unimer micelles due to its structural simplicity and the absence of a strong autofluorescence signal. In fact, collagen fibers are detected owing to their intrinsic SHG signal, which falls in the blue channel when the sample is excited at 820 nm (see Figure S10 for further proofs of the SHG phenomenon, promoted with different excitation wavelengths), while the emission of C153 and the weak tissue autofluorescence are visualized in the green channel. A superposition of the three channels spectral ranges together with the spectrum profile of collagen SHG and the emission of C153-loaded unimer micelles in water is reported in Figure S11.

**Figure 6:**
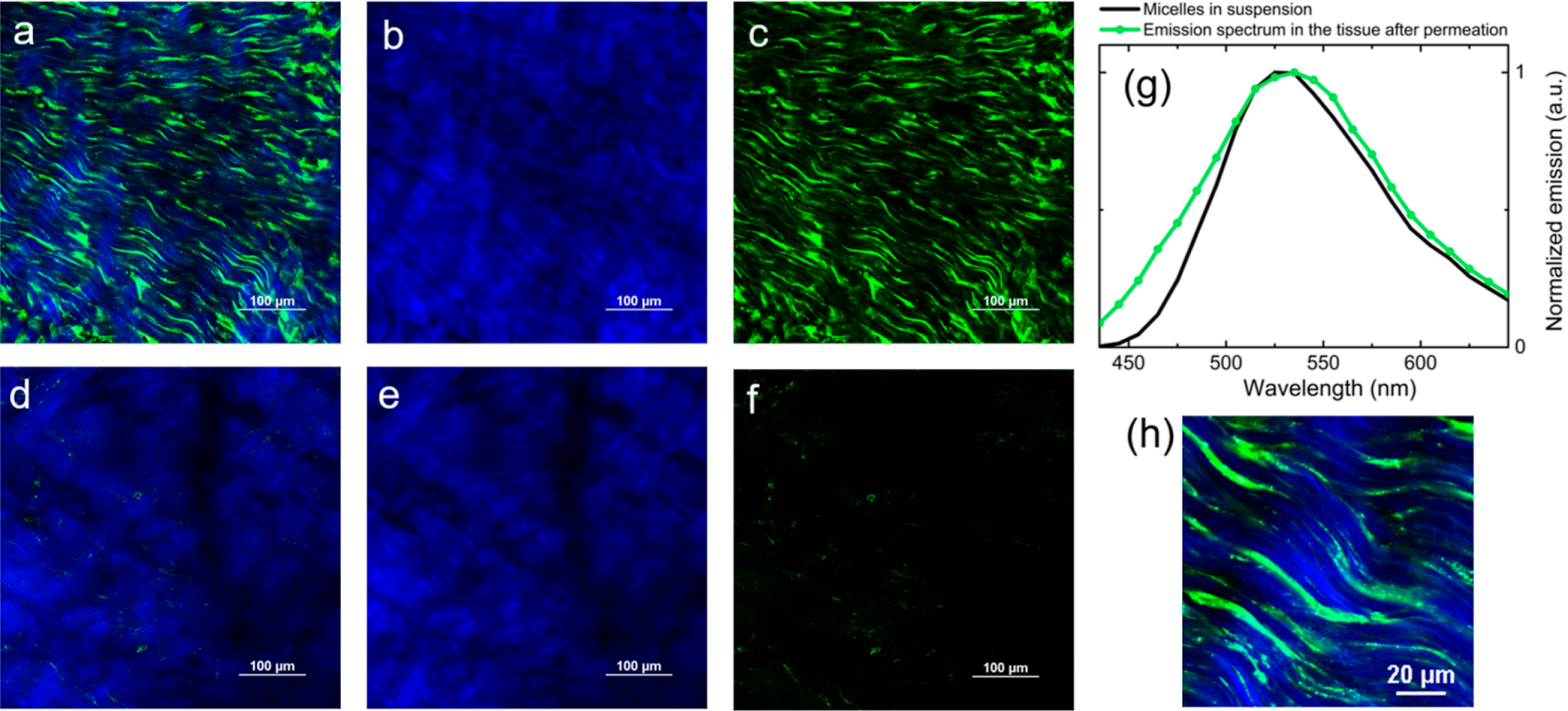
(a–c,h) Sclera treated with unimer micelles in saline solution loaded with C153 (50 μm from surface): (a) channels overlay, (b) blue channel, (c) green channel, and (h) channels overlay of a zoomed region of the tissue (120 μm × 120 μm). (d–f) Sclera treated with saline solution: (d) channels overlay, (e) blue channel, and (f) green channel. All the images were acquired exciting the sample at 830 nm and using the same detector gains. (g) Comparison between the emission spectra (excitation wavelength: 830 nm) acquired in correspondence of panel (a) focal plane and the emission spectrum of an aqueous suspension of unimer micelles loaded with C153. More images acquired from the stained and blank tissues are reported in Figure S12, together with the corresponding emission spectra.

Figure 6a–c reports 2PM images of sclera treated with dye-loaded unimer micelle solution (for comparison, Figure 6d–f reports 2PM images of non-stained sclera). The strong signal detected in the green channel (Figure 6a,c) is mainly attributed to the emission of C153, as confirmed by the emission spectrum (green line in Figure 6g), that is almost superimposable to the emission acquired from the C153-loaded unimer micelles in aqueous suspension (black line in Figure 6g). The superimposition of the spectra in aqueous suspension and in the tissue suggests that the local environment of the dye is not significantly affected during the permeation. Unimer micelles permeate the sclera through the interfibrillar spaces since the signal from C153 arises mainly from the pores within the bundles of collagen fibers (Figure 6h), in a similar way to what was previously described for tocopherol polyethylene glycol 1000 succinate (TPGS) micelles.^64^

**Figure 7:**
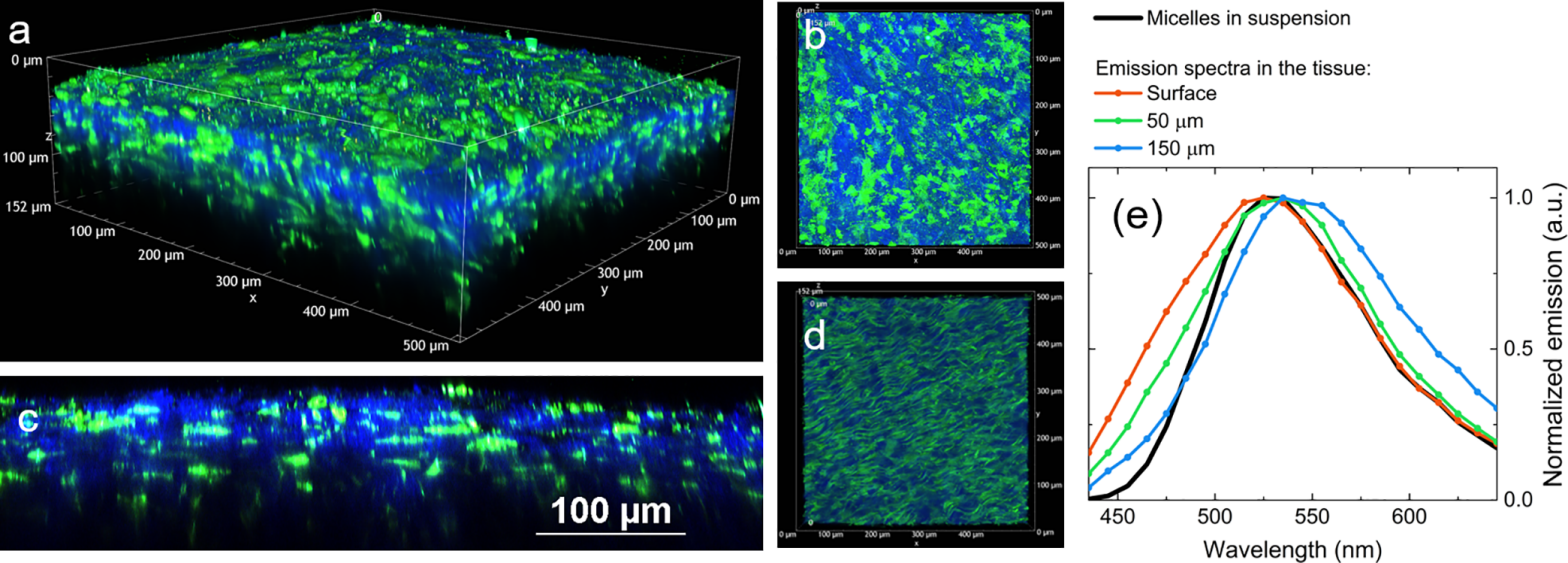
(a–d) Volume rendering of porcine sclera treated with unimer micelles in saline solution loaded with C153, reconstruction from Z-stack (Z-step: 1 μm, total depth: 152 μm): (a) 3D overview, (b) *XY* view, (c) *XZ* slice (512 μm × 152 μm), and (d) *-XY* view. All images are acquired exciting the sample at 830 nm and using the same detector gains. (e) Comparison between the emission spectra acquired in correspondence of different depths (reported in the legend, λ_exc_ = 830 nm) and the emission spectrum of an aqueous suspension of unimer micelles loaded with C153.

In order to have a complete overview of the dye distribution inside the tissue, a Z-scan was acquired on the stained sclera (Figure 7) and for comparison on the plain, untreated sclera (Figure S13). The efficiency of unimer micelles as a nanocarrier for the fluorescent probe C153 is demonstrated by the dye distribution within the tissue (Figure 7a,d): an intense signal is detected in the green channel up to 150 μm in depth, whose spectrum corresponds to the fluorescence of C153, as reported in Figure 7e. A control experiment was performed by treating the sclera with a solution of C153 in water (2 h permeation) in the absence of unimer micelles. Images are reported in Figure S13: adopting the same experimental conditions, the signal from a solution of C153 in water is negligible in the absence of nanocarriers. This control confirms the positive role of unimer micelles as nanocarrier to promote the solubilization of a hydrophobic dye and its permeation across the biological tissue, as well as to preserve the emissive properties of the fluorescent dye.

The detected fluorescence signal decreases rapidly at depths higher than 100 μm (Figure 7c) when the power of the excitation beam is kept constant, mainly due to scattering. However, the fluorescence signal of C153 is detected also from the opposite side of the sample, the choroidal interface (Figure S14), suggesting that C153 permeates through the whole sample in a relatively short time. In any case, no C153 was detected within the receiving solutions at the end of the experiment (see also Section S2.5 of Supporting Information).

### 3.4 Cytotoxicity Studies

**Figure 8:**
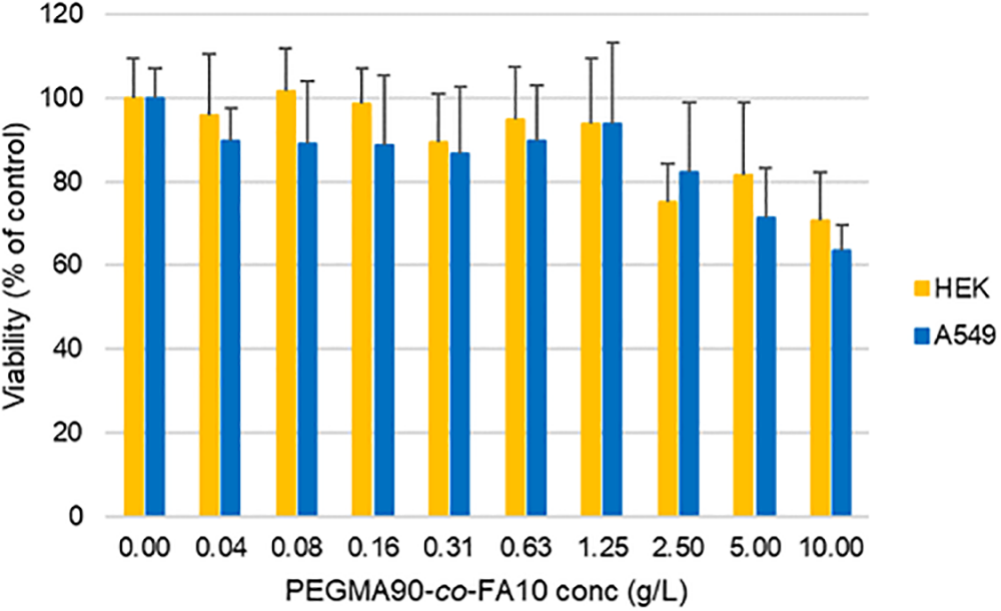
Cell viability of HEK (yellow) and A549 (blue) cultured with several concentrations of PEGMA-*co*-FA10 for 3 h.

Since the unimer micelles were intended for bioimaging following topical application, their cytotoxicity profile in vitro was assessed. Particularly, HEK 293^70^ and A549^71^ cell lines, commonly used in the cytotoxicity assessment, were chosen. After 3 h of contact with PEGMA-*co*-FA10, the MTT assay showed that cell viability was over 80% for concentrations up to 1.25 g/L in both cell lines, as reported in Figure 8.

By increasing the polymer concentration up to 10 g/L, viability slightly decreased but never below 60% with respect to the control. Results here presented are in agreement with previously collected data, showing the non-cytotoxic character of structurally similar PEGylated/fluoroalkyl (meth)acrylic polymers toward Balb/3T3 clone A31 cells^24^ and NIH 3T3 mouse embryonic fibroblast cells and human umbilical vein endothelial cells (HUVECs),^72^ respectively.

Bearing in mind the safety profile of the PEGMA90-*co*-FA10, a precautionary concentration of the polymer should be set below 1.25 g/L. This concentration is compatible with the preparation of C153-loaded unimer micelles having the required spectroscopic properties for working as an efficient fluorescent probe.

## 4 Conclusions

In this paper, we reported the preparation, the characterization, and the application of dye-loaded unimer micelles as fluorescent probes for bioimaging in topical applications. C153-loaded micelles were successfully prepared with the thin-film rehydration procedure, obtaining a suspension of fluorescent unimer micelles by applying a very simple procedure. The effective embedding of the C153 dye within the unimer micelles is demonstrated by steady state and time-resolved anisotropy, showing a different response of the dye in solution and in suspension. Solvatochromic studies suggest that the dye is located in an environment less polar than water, although still quite polar and typical of non-hydrated PEG. The comparison of absorption and emission spectra of C153 in organic solvents, in water and in water suspension of unimer micelles confirms the positive role of micelles in solubilizing C153 in water. At the same time, thanks to the presence of unimer micelles, the good emissive properties of C153 are preserved also in the water environment. Multiphoton microscopy studies demonstrate the efficiency of C153-dye-loaded unimer micelles as fluorescent probes for the visualization of biological tissue in depth. The fluorescent unimer micelles investigated in this work are of interest for ex vivo experiments in topical applications (i.e., eye and skin tissues), where the penetration of the probe in the tissue is an issue. However, the same system is of potential interest also for in vitro experiments with cell cultures. The cytotoxicity studies demonstrated the biocompatibility of the nanomaterial for bioimaging purposes. To the best of our knowledge, this work represents the first attempt of employing non-covalently crosslinked, self-folded unimer micelles as nanocarriers for fluorescent dyes for application in bioimaging. This paper provides a proof of concept of the ability of unimer micelles to efficiently load small hydrophobic organic molecules, which can be easily extended to other molecules and/or applications.

## Supporting Information Available

NMR spectra, GPC analysis, DLS data, additional spectroscopic characterization, and bioimaging data (PDF)
