# Supplementary material for: Amphiphilic Fluorinated Unimer Micelles as Nanocarriers of Fluorescent Probes for Bioimaging
Source: ACS Appl Nano Mater. 2023 Aug 28;6(17):15551–62. doi: 10.1021/acsanm.3c02300 (PMC10496108; doi:10.1021/acsanm.3c02300)
Supplement: Supplementary file 1 — an3c02300_si_001.pdf [file an3c02300_si_001.pdf]

Supporting Information

Amphiphilic Fluorinated Unimer Micelles as Nanocarriers of Fluorescent Probes for Bioimaging

Andrea Delledonne [a], Elisa Guazzelli [b], Silvia Pescina [c], Annalisa Bianchera [c], Giancarlo Galli [b], Elisa Martinelli [b,d,], Cristina Sissa [a,*]*

[a] Dipartimento di Scienze Chimiche, della Vita e della Sostenibilità Ambientale, Parco Area delle Scienze 17A, Università di Parma, 43124 Parma, Italy

[b] Dipartimento di Chimica e Chimica Industriale, Università di Pisa, 56124 Pisa, Italy

[c] ADDRes Lab, Department of Food and Drug, University of Parma, Parco Area delle Scienze 27A, 43124 Parma, Italy

[d] Centro per la Integrazione Della Strumentazione Dell'Università di Pisa (CISUP), Lungarno Pacinotti 43/44, 56126, Pisa, Italy

TABLE OF CONTENTS

1. Physicochemical Characterization 2
  1.1 NMR spectra 2
  1.2 Gel Permeation Chromatography analysis 3
  1.3 DLS data 3
  1.4 Absorption spectra at different C153 loadings 5
  1.5 Overtime stability 6
  1.6 Fluorescence lifetimes data 7
  1.7 Additional spectroscopic measurements 8
  1.8 Fitting of time-resolved fluorescence anisotropy 10
2. Bioimaging 12
  2.1 Second Harmonic Generation from collagen fibers 12

2.2 Detectors spectral range 13
2.3 Images and emission spectra at different depths 14
2.4 Z-scan comparison 15
2.5 Tissue analyzed on the choroidal side 16

1. Physicochemical Characterization

1.1 NMR spectra

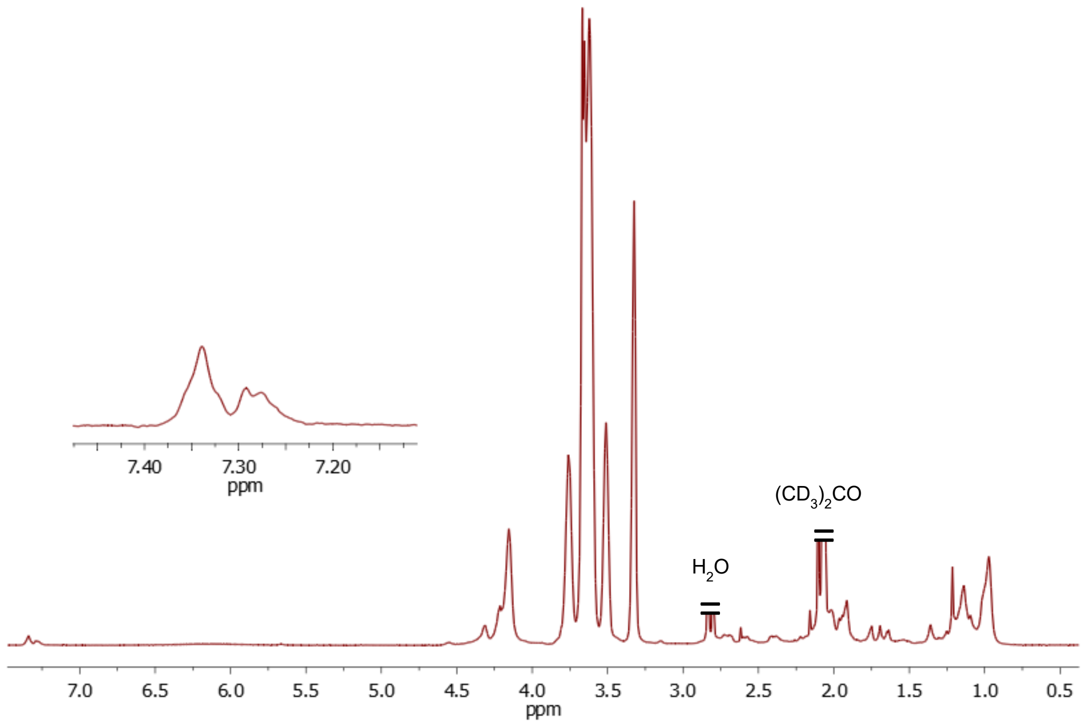

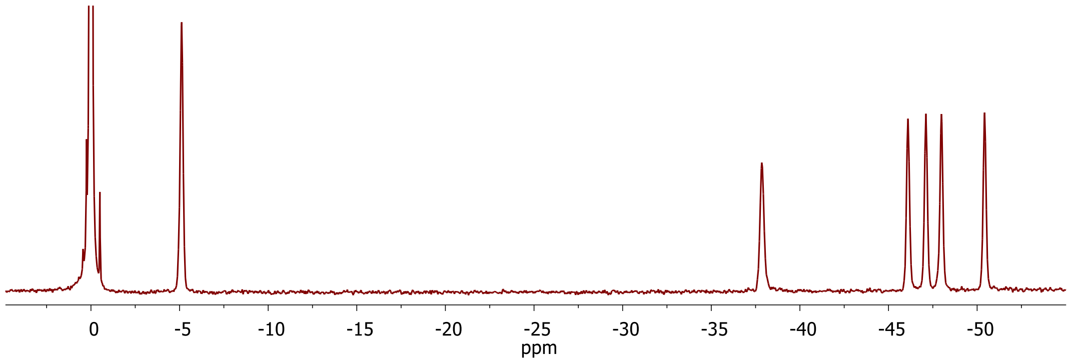

Figure S1. ^{1}H NMR (top) and ^{19}F NMR (bottom) spectra of PEGMA90-co-FA10 in acedone-d_6 and $CDCl_3/CF_3COOH$ respectively.

1.2 Gel Permeation Chromatography analysis

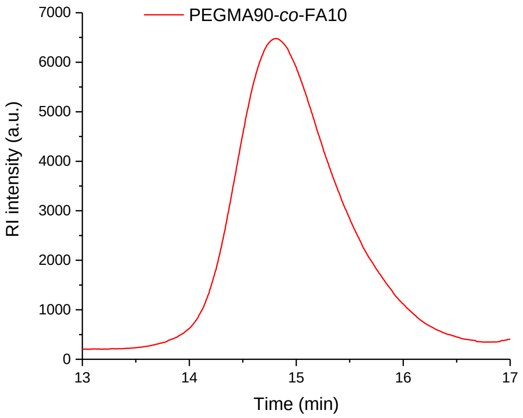

Figure S2. GPC curve of PEGMA90-co-FA10 in $CHCl_3$.

1.3 DLS data

Data were collected at 25 °C as the average of 3 measurements, evaluating the hydrodynamic diameter of the unimer micelles population from the mean size value of the corresponding peak in the intensity distributions.

The population with D_h ~ 5 nm has been observed in the size distributions of all the prepared unimer micelles suspensions (Figure S3 and Table S1), independently from the concentration of C153 or from the presence of NaCl. The population of smaller particles (ascribed to unimer micelles) is the only peak present in the volume and number distributions.

Table S1. DLS results of PEGMA90-*co*-FA10 suspensions.

Sample	Peak 1 mean hydrodynamic diameter (nm)	Peak 1 area intensity (%)	Peak 2 mean hydrodynamic diameter (nm)	Peak 2 area intensity (%)	Peak 1 area volume (%)	Peak 2 area volume (%)
**Saline solution with C153 (15 μM)**	137.8	72.7	5.23	27.3	0	100
RSD %	4.16	0.346	1.23	0.921	0	0
**Distilled water**	131.1	30.5	5.31	69.5	0	100
RSD %	1.34	0.581	3.74	1.33	0	0
**Distilled water with C153 (15 μM)**	119.3	94.5	4.91	5.5	0	100
RSD %	1.3	0.106	2.32	1.82	0	0
**Distilled water with C153 (direct addition, 5 μM)**	101.7	81.9	5.23	18.1	0	100
RSD %	1.05	0.0705	1.13	0.318	0	0
**Distilled water with C153 (direct addition 30 μM)**	144.3	86.6	4.79	13.4	0	100
RSD %	0.703	0.133	0.85	0.864	0	0

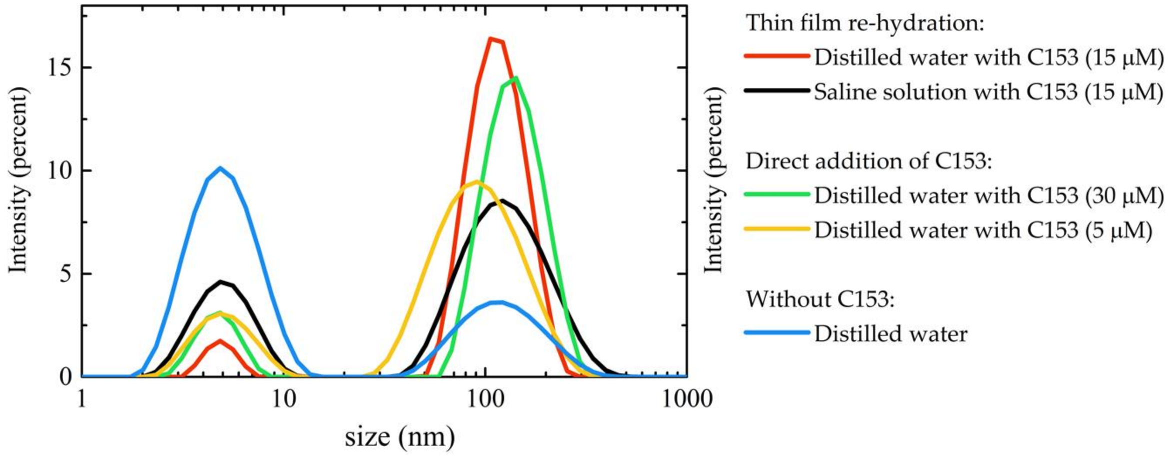

Figure S3. Intensity size distributions of unimer micelles (total PEGMA90-*co*-FA10 concentration: 5 g/L) in different environments and with different C153 loadings.

1.4 Absorption spectra at different C153 loadings

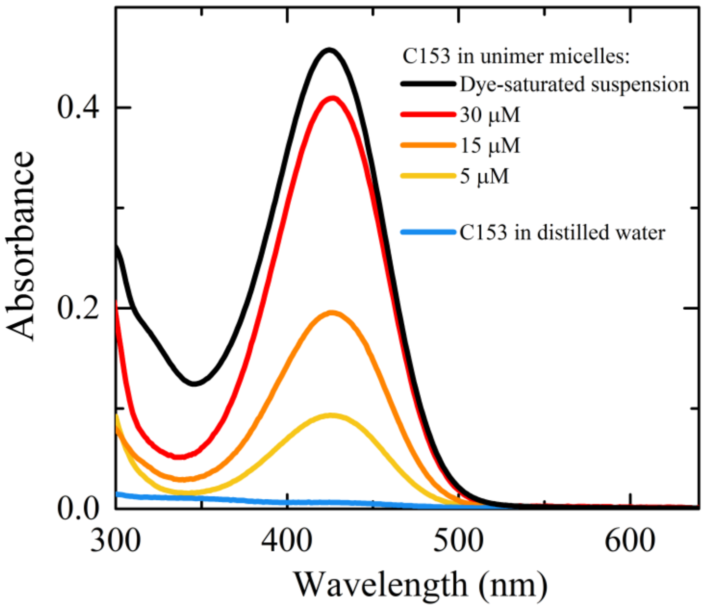

Figure S4. Comparison between the absorption spectra of C153-loaded unimer micelles in distilled water with four different dye concentrations and the absorption of a saturated C153 solution in distilled water. Both the unimer micelles suspension saturated with C153 (0.5 mg/mL) and the one loaded with 15 µM C153 concentration were prepared with the thin-film rehydration methodology reported in the main text, while the 5 µM and 30 µM suspensions were obtained by the direct addition procedure, i.e. by dissolving PEGMA90-co-FA10 in distilled water and adding a small amount of a concentrated C153 solution in acetone (final acetone concentration in the suspension: ~0.1% v/v). All the unimer micelles suspensions were prepared with a total polymer concentration of 5 g/L.

The reported C153 concentrations are theoretical values, since they don't consider eventual losses during the filtration process. The encapsulation efficiency of C153 in the unimer micelles suspensions, especially in the case of the one saturated with the dye, could be increased modifying the preparation protocol, i.e., prolonging the magnetic stirring process for more than 1 hour.

1.5 Overtime stability

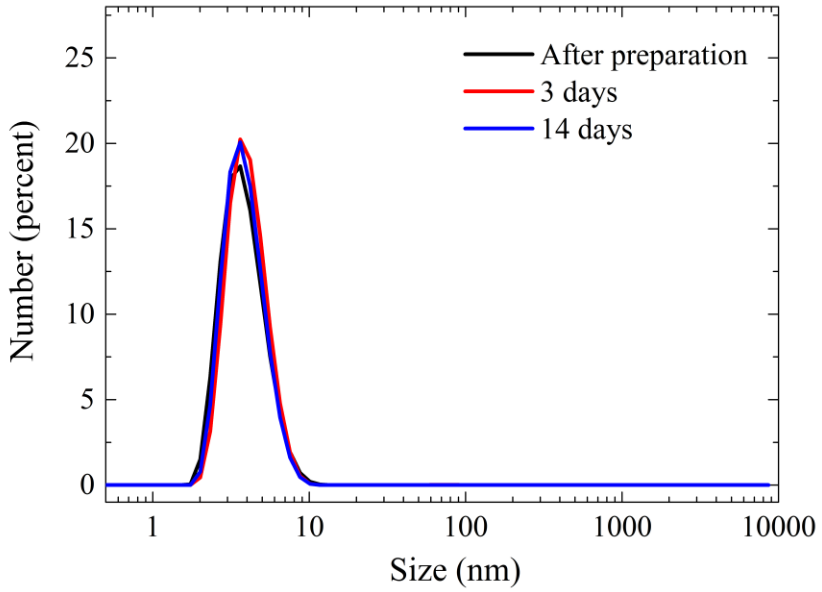

Figure S5. Size distributions by number of C153-loaded PEGMA90-co-FA10 suspension in saline solution acquired after different time periods, up to 14 days from preparation.

1.6 Fluorescence lifetimes data

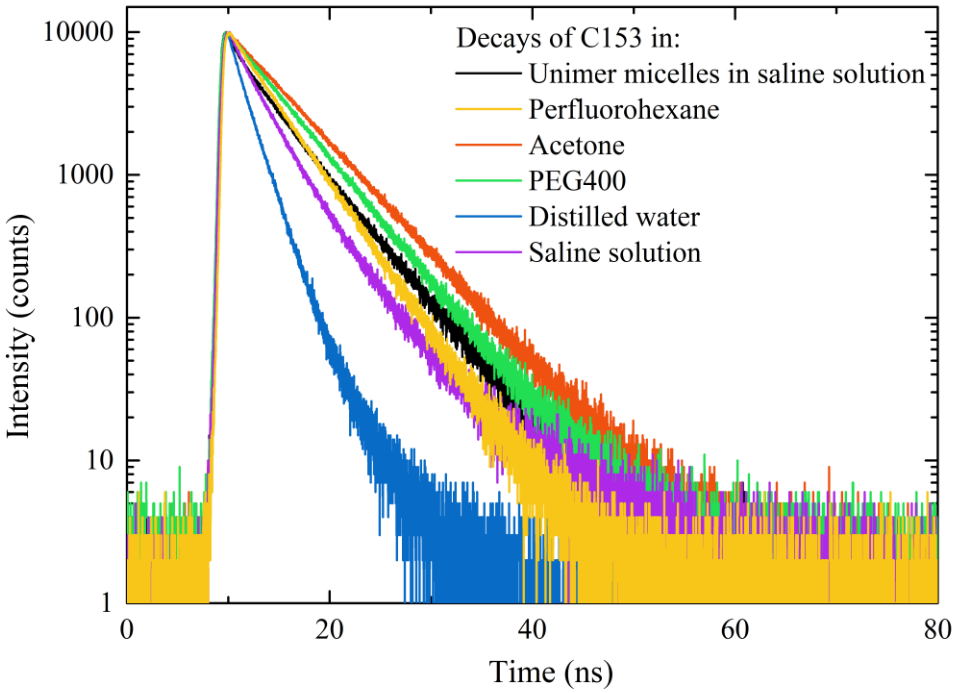

Figure S6. Emission decays of C153 in different environments: perfluorohexane (yellow line λ_{em}= 440 nm) acetone (orange line λ_{em}= 515 nm), PEG400 (green line, λ_{em}= 515 nm), unimer micelles in aqueous suspension (black line, λ_{em}= 515 nm), distilled water (blue line, λ_{em}= 550 nm) and saline solution (purple line, λ_{em}= 515 nm).

Table S2. Exponential components analysis obtained from reconvolution fits of C153 emission decays in different environments.

Sample	τ_1 (ns)	B_1	τ_2 (ns)	B_2	$<\tau>$* (ns)	χ^2
C153 loaded unimer micelles in saline solution	1.768	0.0110	5.000	0.0183	4.433	0.934
C153 loaded unimer micelles in distilled water	2.016	0.0072	5.055	0.0182	4.641	0.919
C153 in distilled water	1.672	0.0257	3.610	0.0013	1.863	0.966
C153 in saline solution	2.5	0.0173	5.243	0.0044	3.454	1.099
C153 in PEG400	4.858	0.0253	9.084	0.0003	4.950	1.073
C153 in acetone	5.556	0.0234	-	-	5.556	0.943
C153 in perfluorohexane	4.058	0.0302	-	-	4.058	0.936

* Average lifetime: $<\tau> = \frac{B_1\tau_1^2 + B_2\tau_2^2}{B_1\tau_1 + B_2\tau_2}$

1.7 Additional spectroscopic measurements

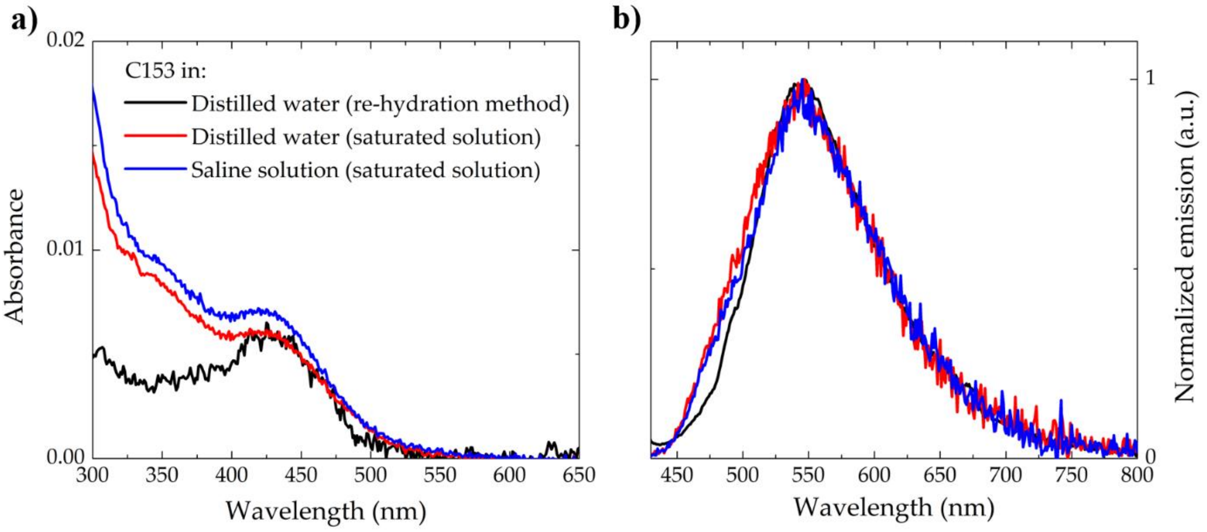

Figure S7. Panel a: comparison between the absorption spectra of C153 in distilled water or saline solution, prepared as described in the experimental section of the main text. Panel b: comparison between the corresponding normalized emission spectra.

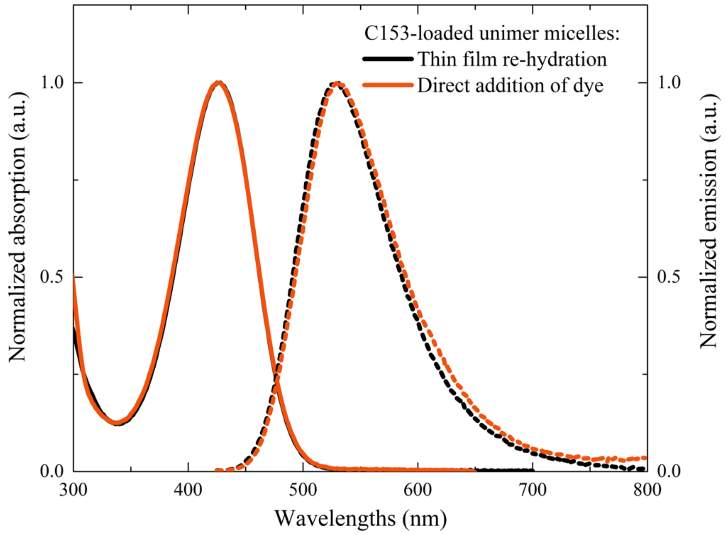

Figure S8. Comparison between normalized absorption and emission spectra of C153-loaded unimer micelles in saline solution (obtained with the thin film re-hydration methodology, total dye concentration: 15 μM) and in distilled water (obtained by the direct addition procedure, total dye concentration: 30 μM). Both suspensions are characterized by a polymer concentration of 5 g/L.

1.8 Fitting of time-resolved fluorescence anisotropy

The parallel and perpendicular fluorescence decays were fitted simultaneously to the following equations:[1]

$$I_{parallel}(t) = \frac{1}{3} I(t)[1 + 2r(t)] \quad \text{(S1)}$$

$$I_{perpendicular}(t) = \frac{1}{3} I(t)[1 - r(t)] \quad \text{(S2)}$$

where r(t) is the anisotropy decay expressed as a bi-exponential function (equation (3) in the main text) and I(t) is the total intensity decay function given by equation (S3):

$$I(t) = I_0[\alpha e^{-\frac{t}{\tau_1}} + (1-\alpha) e^{-\frac{t}{\tau_2}}] \quad \text{(S3)}$$

τ_1 and τ_2 have been fixed to the time constants obtained from the fitting of the total fluorescence intensity decay of C153 loaded unimer micelles in saline solution reported in Figure S6 and Table S1, while α represents the fractional intensity associated with τ_1.

The curves resulting from the fitting are reported in Figure S9 together with the weighted residual distributions, while the optimized fitting parameters are shown in Table S3.

Before starting with the fitting procedures, the parallel decay has been corrected by the G factor of our experimental set-up. Apart from the visual check of randomness of the residual distributions and the calculation of the corresponding χ^2, an additional criterion has been applied to confirm the goodness of fit: good agreement between the experimental value of steady-state anisotropy (r_{ss}) and the one calculated from the optimized fitting parameters using the following equation:

$$r_{ss} = r_0[\beta \frac{\tau_{slow}}{\langle\tau\rangle + \tau_{slow}} + (1-\beta) \frac{\tau_{fast}}{\langle\tau\rangle + \tau_{fast}}] \quad \text{(S4)}$$

The resulting r_{ss} of 0.158 is in line with the experimental value (0.14).

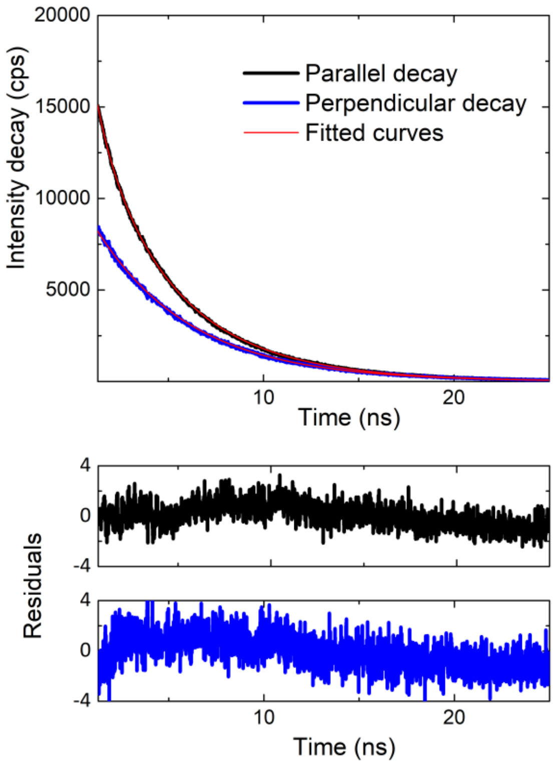

Figure S9. Above: parallel and perpendicular fluorescence decay profiles of C153 loaded unimer micelles measured by exciting the sample at 405 nm and detecting emission at 500 nm. The corresponding fitting curves are showed as overlayed red lines while the weighted residual distributions are reported in the two panels below. The estimated values of χ^2 for the fitting of the parallel and perpendicular components are 0.99 and 1.86, respectively.

Table S3. Optimized parameters obtained from the simultaneous fitting of parallel and perpendicular fluorescence decays.

Fitting	τ_{slow} (ns)	τ_{fast} (ns)	β	r_0
Fluorescence decays	6.90	0.890	0.690	0.334

2. Bioimaging

2.1 Second Harmonic Generation from collagen fibers

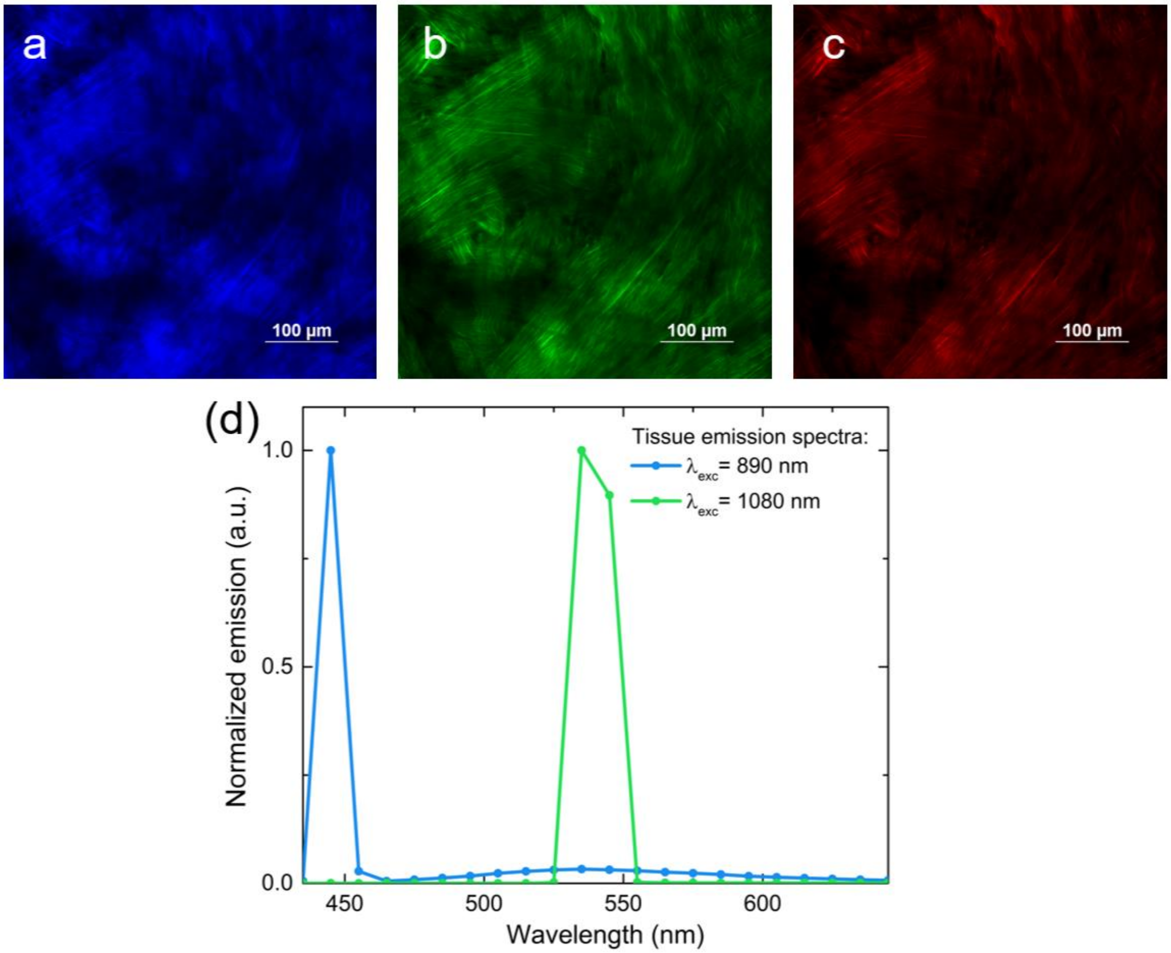

Figure S10. SHG images of scleral collagen: a) 890 nm, b) 1080 nm, c) 1300 nm. In panel (d) are reported the spectrum profiles corresponding to images a) and b).

2.2 Detectors spectral range

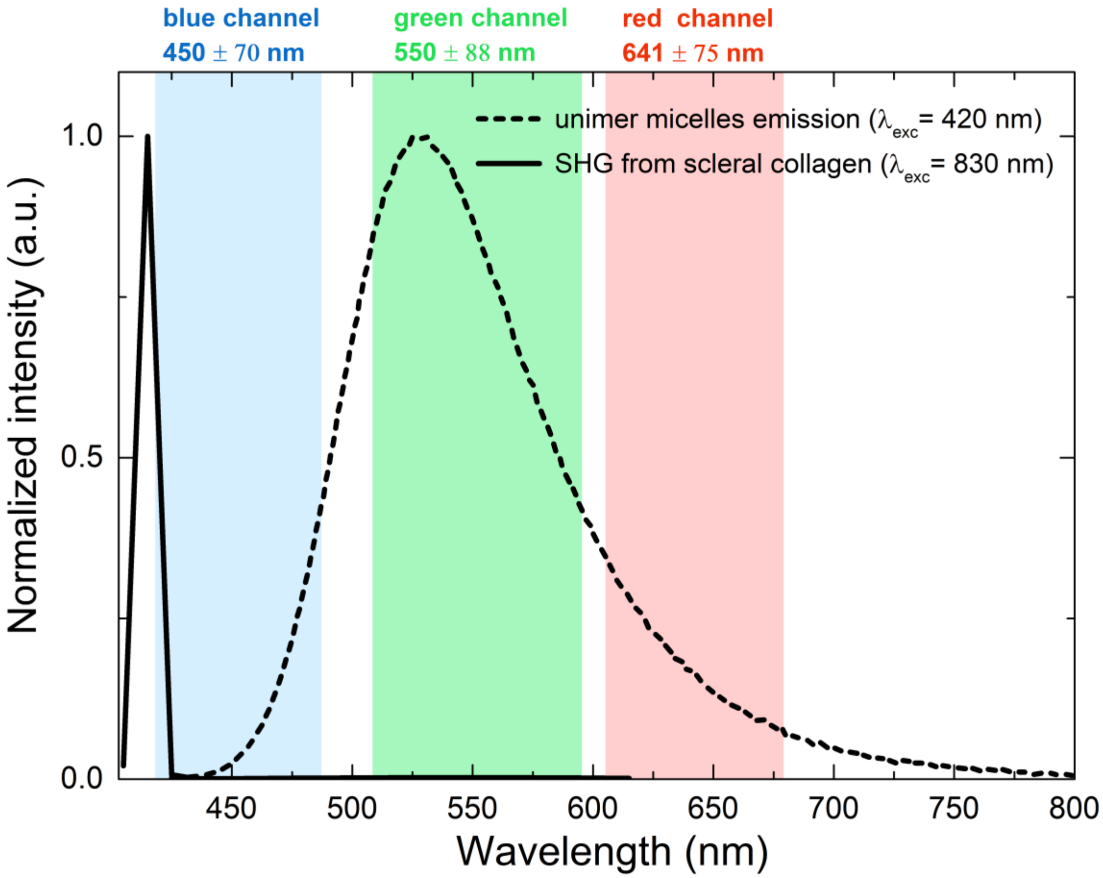

Figure S11. Spectral ranges of the three GaAsP detectors used to acquire the multiphoton microscopy images. The SHG signal from the scleral collagen and the emission of the unimer micelles loaded with C153 are also shown (continuous and dashed black lines, respectively). The excitation wavelength is reported in the legend.

2.3 Images and emission spectra at different depths

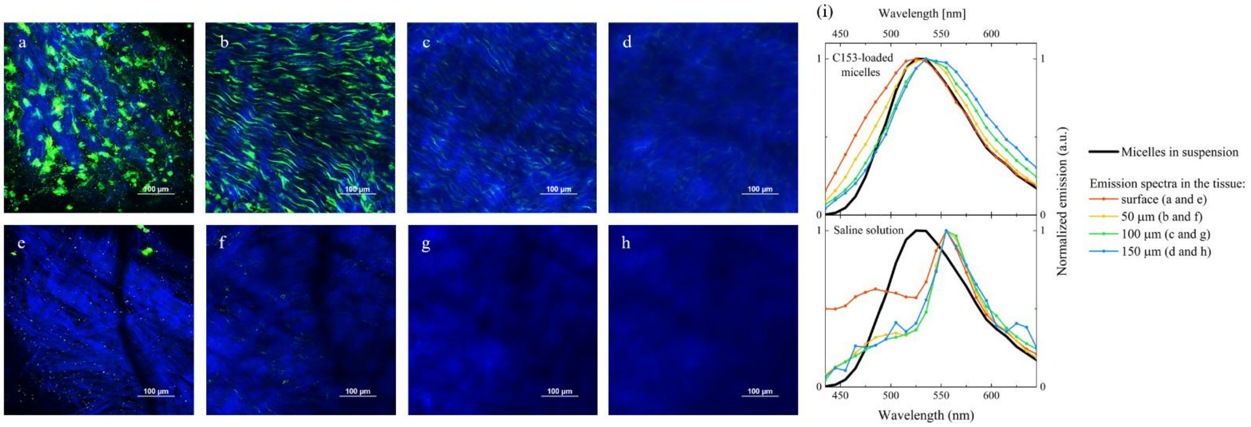

Figure S12. Panels a-d: sclera treated with unimer micelles in saline solution loaded with C153. a) surface, b) 50 μm below the surface, c) 100 μm below the surface, d) 150 μm below the surface. Panels e-h: sclera treated with saline solution: e) surface, f) 50 μm below the surface, g) 100 μm below the surface, h) 150 μm below the surface. The emission spectra collected from the focal planes of the images in panels a-h are reported in panel i. All the images and spectra have been recorded with an excitation wavelength of 830 nm. The images of the two tissues collected at the same depth have been recorded with the same detector gains.

2.4 Z-scan comparison

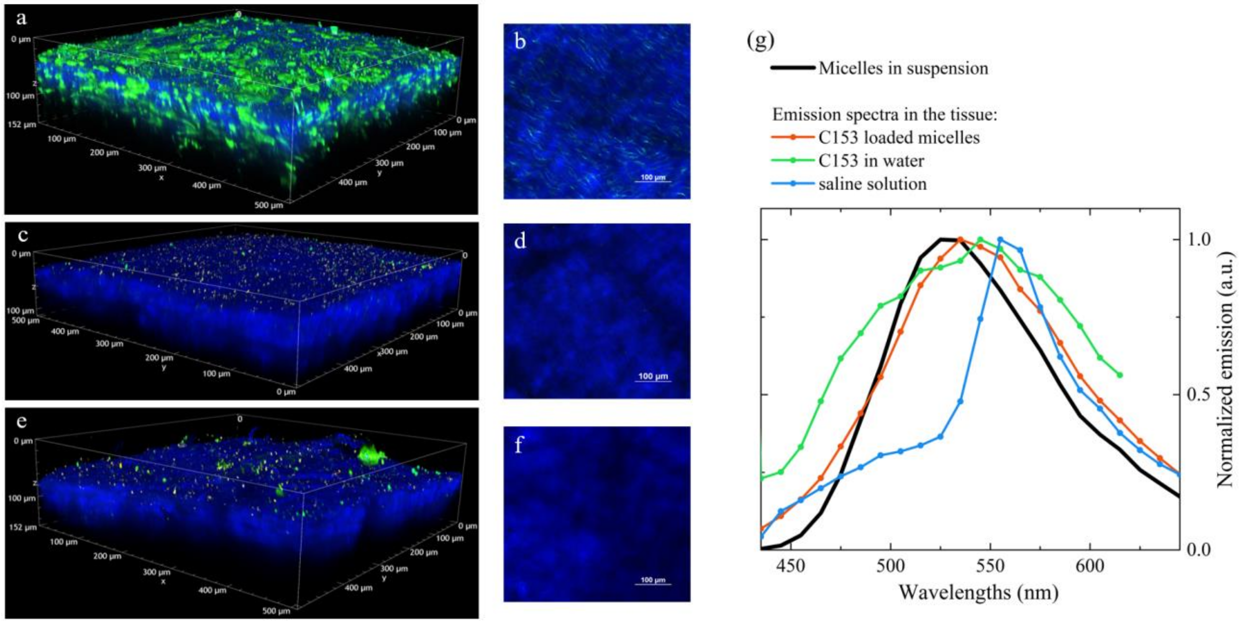

Figure S13. Comparison between Z-scans and images (acquired 100 μm from surface) of: sclera treated with C153-loaded unimer micelles (panels a-b), sclera treated with C153 in distilled water (panels c-d) and sclera treated with saline solution (panels e-f). The emission spectra corresponding to the images b,d and f are reported in panel g.

2.5 Tissue analyzed on the choroidal side

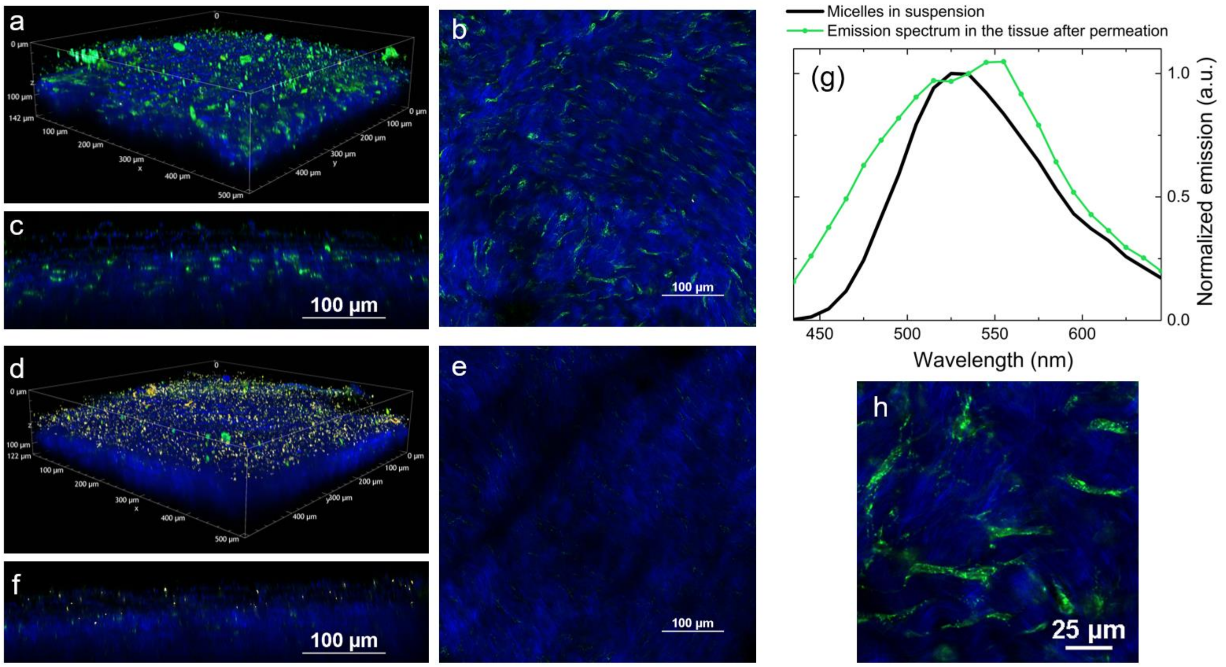

Figure S14. Volume renderings of sclera (choroid side) reconstructed from Z-stacks. Panels a-c, h: sclera treated with unimer micelles in saline solution loaded with C153 (Z-step: 1 µm, total depth: 142 µm): a) 3D overview, b) XY image acquired 50 μm below the surface, c) XZ slice (512 μm x 142 μm) and h) channels overlay of a zoomed region of the tissue (150 μm x 150 μm). Panels d-f: sclera treated with saline solution (Z-step: 1 µm, total depth: 122 µm): d) 3D overview, e) XY image acquired 50 μm below the surface, f) XZ slice (512 μm x 122 μm). All the Images have been acquired exciting the sample at 830 nm and using the same detector gains. Panel g: comparison between the emission spectrum acquired in correspondence of image b) focal plane and the emission of an aqueous suspension of unimer micelles loaded with C153 (excitation wavelength: 830 nm).

The same sample was also visualized on the opposite side, in correspondence of the choroidal interface: the fluorescence signal of C153 is detected also on this side of the tissue (Fig. S14), because a much higher signal in the green channel is recorded in the sample treated with the dye-loaded unimer micelles when compared to the blank one. From Fig. S14h can be noticed that, when C153 reaches this region of the

tissue, the fluorophore tends to accumulate also inside scleral fibroblasts, in contrast to what happens in the external side of the sclera where the dye is mainly localized in the aqueous interfibrillar pores. The emission spectra reported in Fig. S14g confirm the presence of C153 in the choroidal side, but the convolution between the dye emission and the tissue autofluorescence is significant, because only a small amount of nanocarriers was able to fully cross the tissue during the permeation experiment. After the permeation experiments, emission spectra were recorded on the acceptor solutions of the Franz-cells, to assess if the signal of C153 could be detected, but no significant differences have been observed between the solutions in contact with the blank and the stained tissues.
